# Casein kinase I epsilon interacts with mitochondrial proteins for the growth and survival of human ovarian cancer cells

**DOI:** 10.1002/emmm.201101094

**Published:** 2012-06-18

**Authors:** Noah Rodriguez, Junzheng Yang, Kathleen Hasselblatt, Shubai Liu, Yilan Zhou, Jose A Rauh-Hain, Shu-Kay Ng, Pui-Wah Choi, Wing-Ping Fong, Nathalie Y R Agar, William R Welch, Ross S Berkowitz, Shu-Wing Ng

**Affiliations:** 1 Department of Obstetrics/Gynecology and Reproductive Biology, Brigham and Women's HospitalBoston, Massachusetts, USA; 2 School of Medicine, Griffith Health Institute, Griffith UniversityMeadowbrook, Australia; 3 School of Life Sciences, The Chinese University of Hong KongHong Kong, China; 4 Department of Neurosurgery, Brigham and Women's HospitalBoston, Massachusetts, USA; 5 Department of Pathology, Brigham and Women's HospitalBoston, Massachusetts, USA

**Keywords:** casein kinase I epsilon, mitochondria, ovarian cancer, therapeutic target, Wnt signalling

## Abstract

Epithelial ovarian cancer is the leading cause of death among gynaecologic cancers in Western countries. Our studies have shown that casein kinase I-epsilon (CKIε), a Wnt pathway protein, is significantly overexpressed in ovarian cancer tissues and is associated with poor survival. Ectopic expression of CKIε in normal human ovarian surface epithelial cells and inhibition of CKIε in ovarian cancer cells and in xenografts demonstrated the importance of CKIε in regulating cell proliferation and migration. Interestingly, CKIε function did not seem to involve β-catenin activity. Instead, CKIε was found to interact with several mitochondrial proteins including adenine nucleotide translocase 2 (ANT2). Inhibition of CKIε in ovarian cancer cells resulted in suppression of ANT2, downregulation of cellular ATP and the resulting cancer cells were more susceptible to chemotherapy. Our studies indicate that, in the context of ovarian cancer, the interaction between CKIε and ANT2 mediates pathogenic signalling that is distinct from the canonical Wnt/β-catenin pathway and is essential for cell proliferation and is clinically associated with poor survival.

## INTRODUCTION

Epithelial ovarian cancer is the most lethal gynaecologic malignancy among American women with an estimated 14,000 deaths in 2010, thus making it the 5th most common cause of cancer death among women in the United States (Jemal et al, 2010). Over 70% of ovarian cancer cases are diagnosed in the advanced stage of the disease which confers an overall survival of 30% at 5 years (Bast et al, 2002; Kosary, 2008). Standard treatment for advanced ovarian cancer consists of cytoreductive surgery followed by platinum-based chemotherapy and a taxane (McGuire et al, 1996; Ozols et al, 2003). However, chemotherapy related toxicities and drug resistant tumours are significant barriers to treatment. Therefore, the discovery of novel therapeutic targets is important in the battle against this deadly disease. Recent genomic analyses of many human cancers have revealed that a significant number of tumours have alterations in a few core pathways (Luo et al, 2003; McCormick, 1999; Rodriguez-Viciana et al, 2004; Wullschleger et al, 2006). Identifying and characterizing these core pathways provides a foundation for therapeutic development.

Casein kinase 1-epsilon (CKIε) was identified as an important tumour antigen in a recent work in our laboratory using a novel reverse capture antibody microarray technique (Tang et al, 2010). CKIε is one of seven mammalian isoforms of the casein kinase family, a group of ubiquitous and highly conserved serine/threonine-specific kinases, which are involved in signal transduction pathways (Fish et al, 1995; Ko et al, 2002; Peters et al, 1999; Price, 2006). Casein kinases phosphorylate key regulatory proteins in the control of cell differentiation, proliferation, chromosome segregation and circadian periodicity (Knippschild et al, 2005; Ko et al, 2002; Meng et al, 2010). CKIε is a protein product of the CSNK1E gene and has been shown to be important in regulating cell division and tumour growth in human pancreatic adenocarcinoma and salivary gland cancer by phosphorylating key proteins in the Wnt signalling pathway (Brockschmidt et al, 2008; Frierson et al, 2002; Peters et al, 1999; Polakis, 2007; Price, 2006).

The Wnt signalling pathway is composed of canonical and non-canonical pathways. In the canonical Wnt/β-catenin pathway, CKIε phophorylates Dishevelled (Dsh/Dvl) which stabilizes β-catenin. Once stabilized, β-catenin enters into the nucleus and causes activation of gene expression which regulates cell proliferation and differentiation (Gao et al, 2002; Kishida et al, 2001; Polakis, 2007). The non-canonical Wnt pathways are activated by a subset of Wnts and are independent of β-catenin. Neither the molecular frameworks for the non-canonical pathways have been defined nor has the role of CKIε in ovarian cancer and its clinical utility been reported.

In this report, we used *in vitro* ectopic expression of CKIε as well as pharmocologic inhibition and shRNA knockdown of the CKIε expression to reveal that CKIε is critical to cell proliferation and migration. We demonstrate that selective inhibition of CKIε decreased tumour burden *in vivo* and made ovarian cancer cells more susceptible to chemotherapeutic agents. Furthermore, over-expression of CKIε is associated with decreased survival in patients with advanced stage ovarian cancer. Finally, we report a novel interaction between CKIε and a mitochondrial protein, adenine nucleotide translocase 2 (ANT2) in the non-canonical Wnt pathway. These findings strongly suggest CKIε as a promising therapeutic target for the treatment of ovarian cancer.

## RESULTS

### CKIε is overexpressed in ovarian cancer tissue samples and ovarian cancer cell lines

**Figure 1:**
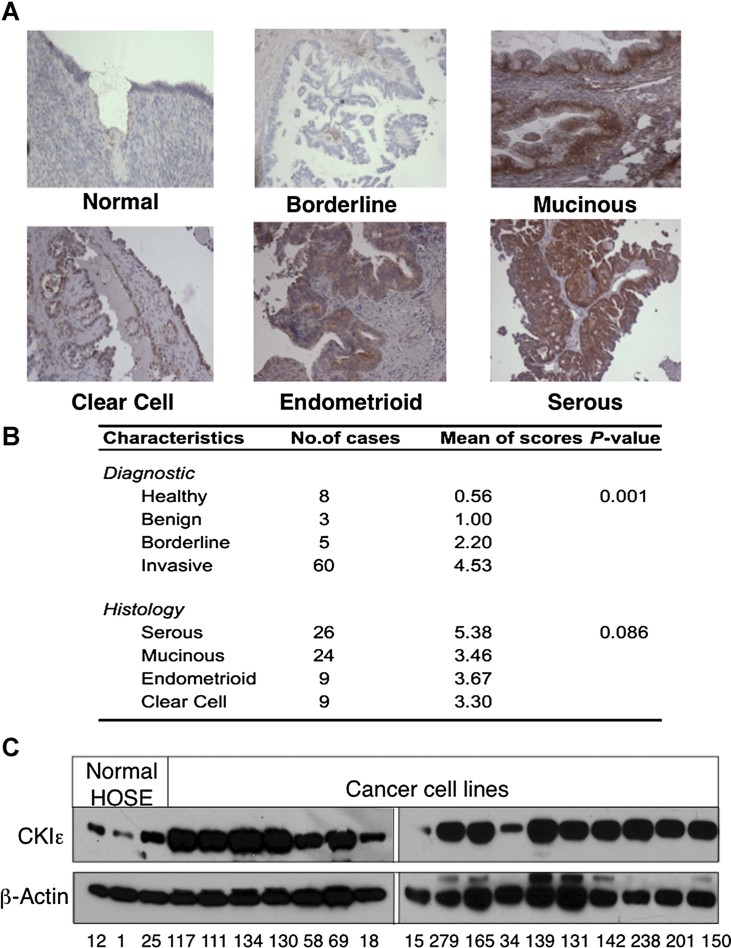
Expression of CKIε in ovarian tumours and cancer cell lines Representative figures of immunohistochemical staining of normal ovaries and ovarian tumour tissues for CKIε using the monoclonal mouse anti-human CKIε antibody from BD Biosciences.Statistical analysis of CKIε staining according to diagnostic and histological characteristics.Western blot analysis of CKIε expression in normal HOSE cells and ovarian cancer cell lines. Quantified CKIε signals presented at the bottom were normalized to signals of the β-actin of the cell lines. From left to right: HOSE 1-15, HOSE624, HOSE80-PC, OVCA810, RMG1, OVCA420, SKOV3, MCAS, DOV13, OVCA680, TOV21G, TOV112D, RMUGS, RMUGL, OVCA3, OVCA429, OVCA432, OVCA433, SKOV3-IP and HeyA8.

To investigate the expression pattern of CKIε in ovarian tissues, we performed immunohistochemistry (IHC) using a monoclonal CKIε antibody on 76 paraffin-embedded ovarian tissue samples. Examples of normal ovarian tissue, borderline tumours and invasive tumours stained for CKIε are shown in Fig 1A. Strong immunoreactivity was found in borderline tumours (mean score = 2.20) and in invasive tumours (mean score = 4.53), which showed a significant difference (*p-*value = 0.001) when compared with healthy and benign ovarian tissues that had a mean score of 0.56 and 1.00, respectively (Fig 1B and Supporting Information Fig S1). There were no significant differences of staining among subtypes of ovarian tumours (*p-*value = 0.086). We have confirmed the specificity of the staining and also compared the staining for CKIε with the closely related CKIδ (Supporting Information Fig S1). While ovarian tumours stained positive for CKIε, another slides of the same tumours showed very weak to negative staining for CKIδ. We have also performed IHC for the more distant CKIα isoform (Supporting Information Fig S1D). This isoform is expressed in almost all types of cells in the ovarian tissues. Unlike CKIε that is not significantly expressed in the normal ovarian surface epithelium, CKIα is expressed significantly in the normal ovarian surface epithelial cells similar to the tumour cells. Figure 1 demonstrates a Western blot analysis revealing CKIε to be overexpressed in 15 of 17 ovarian cancer cell lines, when compared with normal human ovarian surface epithelial (HOSE) cells. Further comparison of CKIε, CKIδ and CKIα expression patterns in ovarian cell lines showed that only one out of eight cancer cell lines had moderate CKIδ expression, two out of eight ovarian cancer cell lines showed overexpression of CKIα relative to the normal HOSE cells (Supporting Information Fig S2). In conjunction with the immunohistochemical data, the Western blot results suggest that while there are different CKI isoforms in the ovarian tumour cells, it is likely that only the CKIε isoform is significantly overexpressed in the majority of ovarian cancer cells relative to the normal ovarian surface epithelial cells.

Characterization of CKIε in breast cancer has identified several somatic mutations in the 5′-coding region of the gene, which could lead to imaginal disc overgrowth in *Drosophila* (Dolezal et al, 2010; Foldynova-Trantirkova et al, 2010; Fuja et al, 2004). We have sequenced the proposed region using DNA from 38 laser-microdissected ovarian tumour samples and genomic DNA from 8 ovarian cancer cell lines. No such somatic mutations were identified in the cancer DNA (Supporting Information Fig S3). Hence, it is apparent that the overexpressed CKIε transcripts in ovarian cancer harbour wild-type sequence in this region.

### Ectopic expression of CKIε increases cell proliferation and spheroid formation *in vitro*

**Figure 2:**
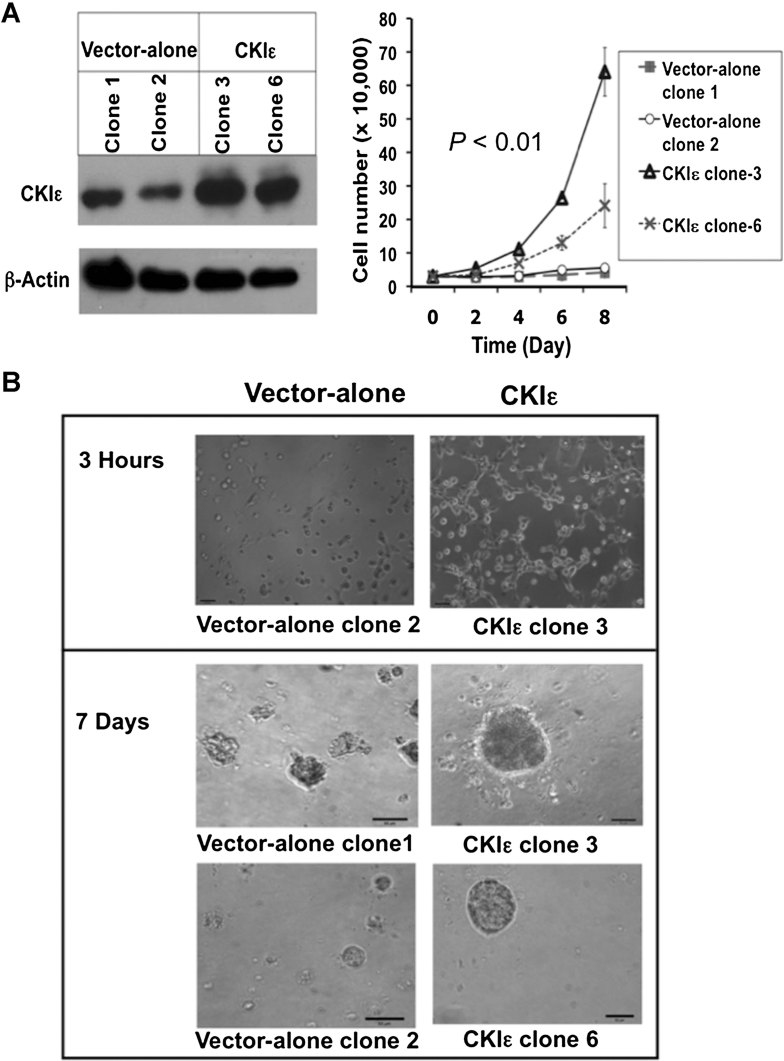
Ectopic expression of CKIε in HOSE cells promotes cell growth and increases spheroid size in Matrigel cultures Western blot analysis using the monoclonal mouse anti-human CKIε from BD Biosciences to show the increased expression of CKIε in the ectopic expression clones compared with the vector transfected clones (left). The growth rates of the four HOSE sublines determined by cell counting (right).Micrographs of ectopic expression clones and vector transfected clones growing in Matrigel culture.

To investigate the effects of CKIε on cell proliferation, we introduced a CKIε-expressing plasmid into the genome of HOSE cells to ectopically express CKIε. Ectopic expression of CKIε in the selected clones was confirmed by Western blot analysis (Fig 2A). HOSE cells which ectopically expressed CKIε (Clones 3 and 6) had higher rates of proliferation compared with control HOSE cells (Clones 1 and 2) (Fig 2A). In addition, CKIε clones 3 and 6 HOSE cells demonstrated a higher propensity to cluster together and form large three-dimensional (3D) spheroids in Matrigel, which were 10 (Clone 6) to 13 (Clone 3) times larger than the spheroids formed by controls (*p* < 0.001) (Fig 2B and Supporting Information Fig S4). These observations suggest that CKIε is important in regulating cell proliferation and promotes large spheroid formation.

### CKIε inhibition suppresses growth of ovarian cancer *in vitro* and *in vivo* and reduces migration capacity

**Figure 3:**
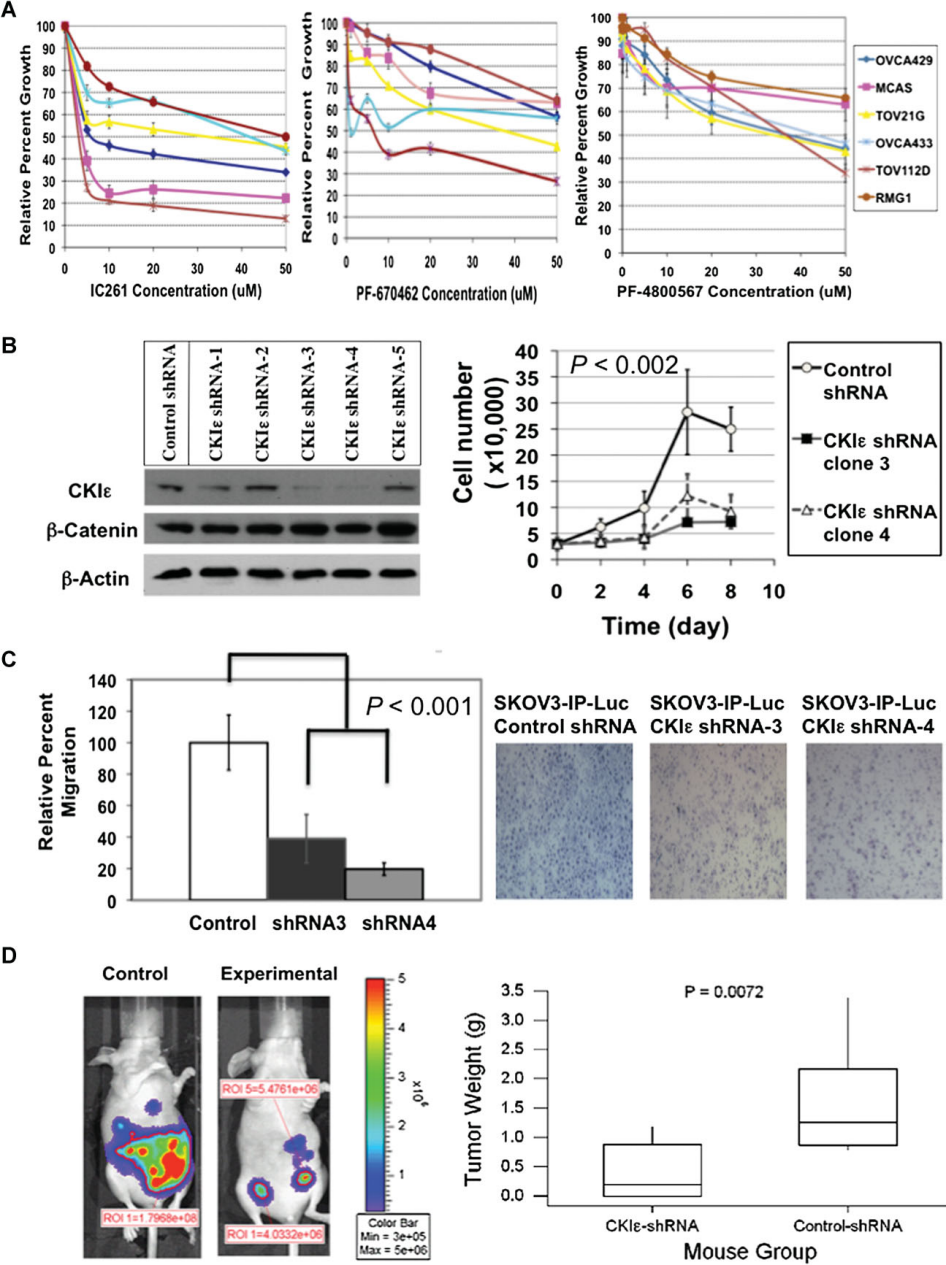
Inhibition of CKIε in ovarian cancer cells suppressed cell proliferation, migration rate and *in vivo* tumour burden MTT assays to investigate the effects of CKIε/δ inhibitors IC261, PF-670462 and PF-4800567 on the proliferation rate of ovarian cancer cell lines. The *p*-values of differences between treated and untreated cells are <0.05 between 10 and 50 µM for IC261, between 20 and 50 µM for PF-670462 and PF-4800567, respectively.Western blot analysis to show the expression levels of CKIε and β-catenin in the ovarian cancer cells harbouring different shRNA constructs. The antibodies were from BD Biosciences. The ovarian cancer cells harbouring, respectively, CKIε shRNA constructs 3 and 4 demonstrated reduced proliferation rates.Migration rates of ovarian cancer cells harbouring control shRNA, CKIε shRNA construct 3 and CKIε shRNA construct 4 measured by transwell assay. Representative figures of the migrated cells on the membranes are shown on the right.Tumour burden as measured by whole body luminescence imaging (left) and excised tumour weights (right). The inter-quarter ranges (IQR) for the dispersion of excised tumour weights were 1.301 for the Control shRNA group and 0.878 for the CKIε shRNA group.

To study the effects of CKIε inhibition on cell growth, three pharmacological inhibitors of CKIδ/ε (IC261, PF-670462 and PF-4800567) were tested in ovarian cancer cell lines. All CKIδ/ε inhibitors significantly reduced the growth rate of ovarian cancer cells, with IC261 showing a more pronounced effect (Fig 3A). As IC261 is far less potent than the other inhibitors in inhibiting CKIδ and CKIε and studies have shown that IC261 triggers the mitotic checkpoint other than CKI inhibition (Behrend et al, 2000; Cheong et al, 2011), we determined cell cycle distribution of two ovarian cancer cell lines, SKOV3 and MCAS, after treatments with doses of the inhibitors that caused about 50% of growth inhibition. IC261 caused a significant parallel depletion of G1 phase and cell cycle arrest in G2/M phase (Supporting Information Fig S5). Both treatments with PF-670462 and PF-4800567 did not show such drastic changes. Hence, it is likely that the pronounced growth inhibition caused by IC261 was due to unwanted side effect other than inhibition of CKIε.

To evaluate the consequence of CKIε inhibition not due to off-target effects, we used lentivirus to introduce short-hairpin RNAs (shRNAs) to specifically target and suppress the CKIε gene expression in SKOV3-IP^Luc^ and MCAS ovarian cancer cells. Similarly, an shRNA construct harbouring a scrambled sequence was introduced into the two cancer cell lines to establish the respective control cell lines. A Western blot analysis confirmed reduced CKIε expression in cell lines harbouring two CKIε-targeting shRNA constructs, Clones 3 and 4 (Fig 3B). We have characterized the MCAS and SKOV3-IP^Luc^ cell lines with CKIε knockdown and the results from these cell lines were very similar. We first studied the impact of suppression of CKIε expression on cell proliferation by comparing the growth rate of Clones 3 and 4 to that of control shRNA cells. Clones 3 and 4 demonstrated significantly decreased growth rates compared to control shRNA cells (*p* < 0.002; Fig 3B). The reduced growth rate correlated directly with reduced CKIε expression.

Next, we used a transwell migration assay to illustrate the influence of selective CKIε inhibition on cell migration. Cells harbouring the CKIε shRNA construct demonstrated a 60–80% reduction in their capacity to migrate compared with control shRNA (*p* < 0.001; Fig 3C). Together, these findings further support that CKIε is crucial for cell growth and migration.

To test our findings *in vivo,* we divided 24 mice into two equal groups and inoculated the control group with SKOV3-IP^Luc^ cells harbouring the control shRNA and the experimental group with SKOV3-IP^Luc^ cells harbouring the CKIε shRNA construct 3. SKOV3-IP^Luc^ cells were used because they have previously been shown to produce peritoneal carcinomatosis in mice, which closely resembles advanced ovarian cancer. After 28 days, bioluminescent images of both groups of mice were taken. Once the images were captured, the mice were sacrificed and the tumours were harvested and weighed. A comparison of the bioluminescent images demonstrated significantly less tumour growth in the experimental group mice compared to the control group mice (Fig 3D and Supporting Information Fig S6A). These findings were confirmed by comparing the weights of the harvested tumours. The CKIε shRNA tumours weighed significantly less compared to the tumours from the control shRNA mice (Median weight is 0.195 g *vs.* 1.25 g, *p* = 0.0072, Fig 3D). Examination of the harvested xenograft tumours showed that the knockdown tumours maintained reduced expression of CKIε, much less robust tumour growth and proliferation rate as represented by weaker Ki67 staining than the control tumours (Supporting Information Fig S6B). The combined *in vitro* and *in vivo* data strongly indicate that CKIε inhibition is capable of decreasing cell growth and reducing tumour burden.

### Co-immunoprecipitation and co-localization of mitochondrial proteins reveals a novel interaction of CKIε with ANT2

**Figure 4:**
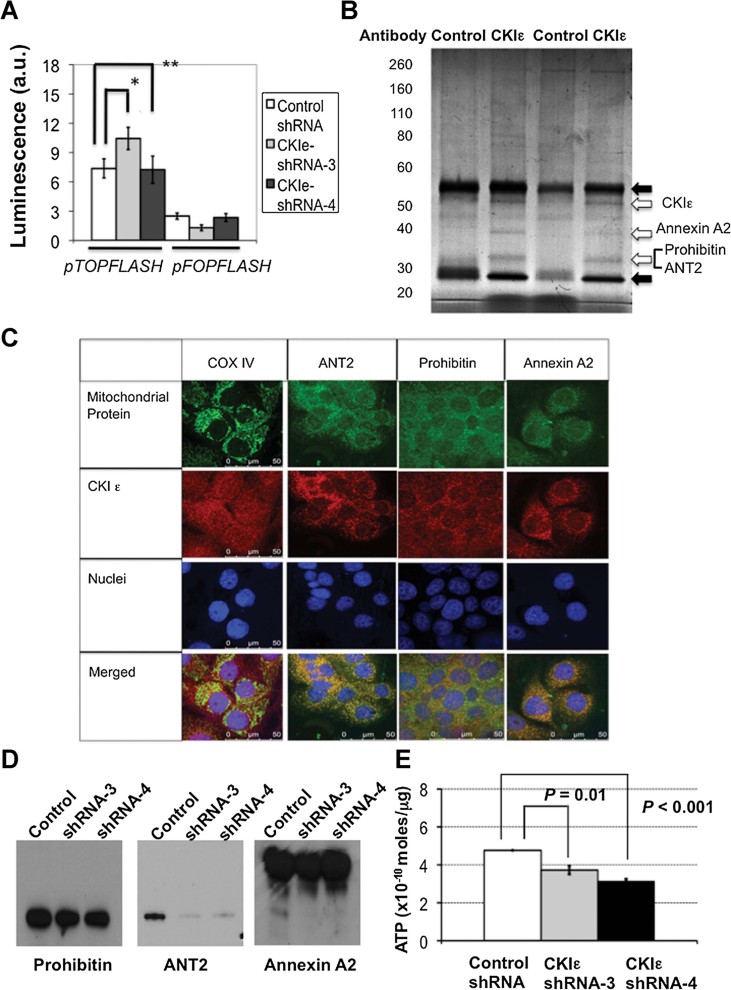
Ovarian cancer cell lines with CKIε knockdown did not show changes in β-catenin activity but reduced expression of mitochondrial protein ANT2 Reporter luciferase assay to evaluate any changes in the β-catenin activity of the MCAS ovarian cancer cells harbouring control and CKIε knockdown constructs as described in Materials and Methods Section. The β-catenin binding elements-harbouring luciferase construct (*pTOPFLASH*) and control luciferase construct (*pFOPFLASH*) used in the transfections are indicated at the bottom. *p = 0.519; **p = 0.734.Colloidal Coomassie universal protein dye-stained gel to show the resolved immunoprecipitated proteins using CKIε antibody (BD Biosciences) and a non-specific mouse control antibody. Lanes of two separate co-immunoprecipitation reactions are shown here. Open block arrows indicate the protein bands that were not present in the control lanes. The heavy chains and light chains of the immunoprecipitation antibodies are marked by solid block arrows.Confocal immunofluorescence of wild-type MCAS cells to demonstrate the co-localization of CKIε and mitochondrial proteins. The fluorescence of mitochondrial proteins were pseudo-coloured as green, whereas the fluorescence of CKIε was pseudo-coloured as red. COX IV was used as marker for intracellular mitochondria.Western blot analysis to show the expression of the three mitochondrial proteins in the control and knockdown cell lines.Measurement of intracellular ATP content in the control and CKIε knockdown cell lines.

CKIε is known to function in the canonical Wnt/β-catenin pathway by stabilizing β-catenin protein (Gao et al, 2002; Kishida et al, 2001; Polakis, 2007). We found that, however, the β-catenin protein level was unchanged in the wild-type and knockdown cancer cell lines (Fig 3B), as well as in the xenograft tumours (Supporting Information Fig S6C). A luciferase reporter assay after transfection of the cell lines with a luciferase expression construct under the control of a promoter harbouring tandem β-catenin-binding elements did not show significant differences in luciferase activity either (Fig 4A). Thus, both *in vitro* and *in vivo* data suggest that the canonical Wnt/β-catenin pathway in ovarian cancer cells was not affected by CKIε inhibition.

To explore other potential mechanisms of CKIε function, co-immunoprecipitation assays were performed to determine whether CKIε was acting alone or in concert with other interacting partners. Gel electrophoresis of co-immunoprecipitates prepared using the anti-CKIε antibody consistently showed three bands of additional proteins when compared with the immunoprecipitates from a non-specific mouse control antibody (Fig 4B). Peptide mass analysis of the excised protein bands identified besides CKIε three mitochondrial proteins Annexin A2, Prohibitin and ANT2 in the CKIε co-immunoprecipitates (Supporting Information Fig S7). To confirm our results, we employed specific antibodies that targeted the three mitochondrial proteins in a reciprocal co-immunoprecipitation assay and the results support the interactions between CKIε and Annexin A2, Prohibitin and ANT2 (Supporting Information Fig S8A). To explore the phosphorylation status of the interacting proteins, we probed CKIε immunoprecipitate with an antibody that recognized proteins with phosphorylated threonine. A protein band at the position of ANT2 and Prohibitin was recognized by this antibody (Supporting Information Fig S8B). To determine whether CKIε and the interacting proteins are located in the mitochondria, confocal immunofluorescent analysis was performed. There were significant amounts of cytoplasmic CKIε proteins that overlapped with the mitochondrial marker, COX IV. Similarly, CKIε showed significant co-localization with the three mitochondrial proteins (Fig 4C). Furthermore, we have also fractionated nuclear, cytosolic and mitochondrial proteins from the cancer cells and performed Western blot analysis of the fractionated proteins (Supporting Information Fig S9). Except for Annexin A2 that was present equally in all fractions, there were significant levels of CKIε, ANT2 and Prohibitin in the mitochondrial fraction. It was also noted that significant amounts of CKIε and ANT2 were also present in the nuclear fraction.

To study the effects of CKIε knockdown on the three mitochondrial proteins, Western blot analysis was performed to compare the levels of these three mitochondrial proteins between the wild-type and knockdown cell lines (Fig 4D and Supporting Information Fig S9). Expression of Prohibitin and Annexin A2 was not significantly different in the CKIε shRNA and control shRNA lysates. However, ANT2 was significantly decreased in the CKIε shRNA lysate compared to the control shRNA lysate. Similar result was also identified in the Western blot analysis of mouse xenografts (Supporting Information Fig S6C). Consequently, the unexpected effect of CKIε on ANT2 warranted further exploration.

ANT2 has been shown to be up-regulated in highly proliferating cancer cells and mediates the exchange of ADP and ATP on the inner mitochondrial membrane (Barath et al, 1999; Dorner & Schultheiss, 2007; Dorner et al, 1997; Houldsworth & Attardi, 1988; Neckelmann et al, 1987). IHC of ANT2 in ovarian tissues showed that the protein is overexpressed in different subtypes of ovarian tumours (Supporting Information Fig S10A). We analysed cellular ATP levels in control shRNA cells and compared them to ATP levels in CKIε shRNA cells. The ATP levels in the CKIε shRNA cells were significantly lower than the ATP levels in control shRNA cells (Fig 4E). These findings confirm that both ANT2's expression and its effect on ATP production are decreased in cells whose CKIε expression has been suppressed, and support a novel interaction between CKIε and ANT2 that is independent of β-catenin.

### CKIε-selective inhibition increases response to chemotherapeutic agents *in vitro*

**Figure 5:**
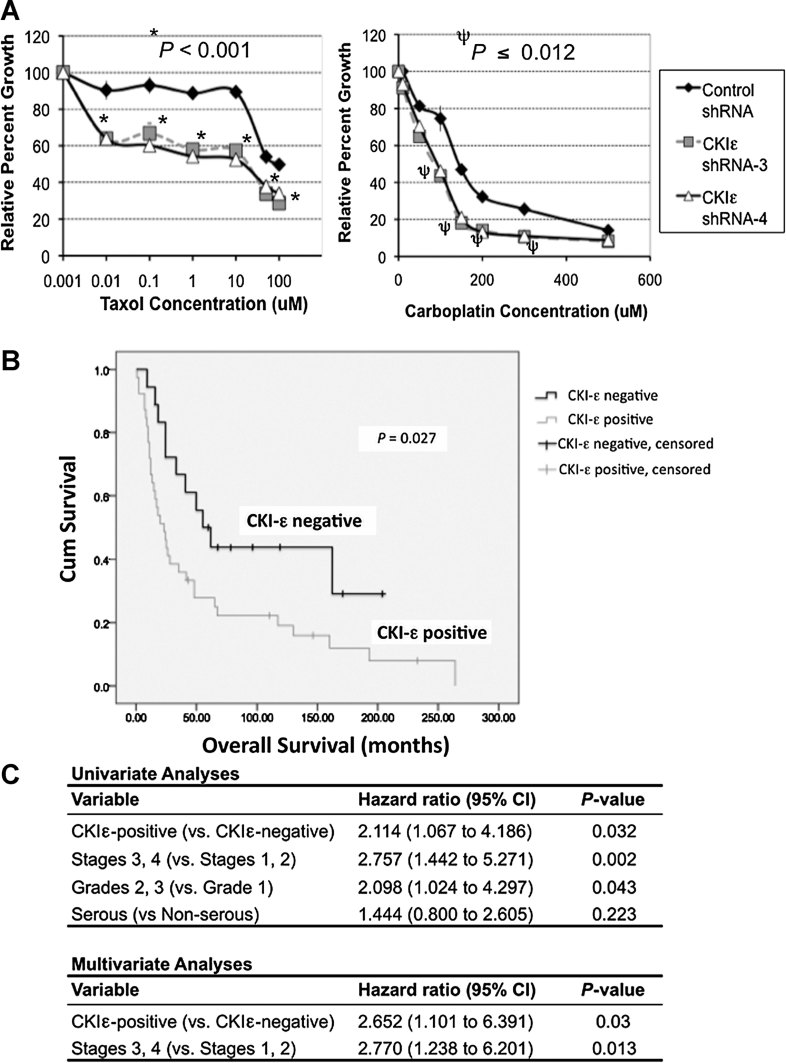
Expression of CKIε in ovarian cancer cells is associated with *in vitro* chemoresponse and poor clinical outcome of ovarian cancer patients The response of control and CKIε knockdown SKOV3 cells to Taxol and carboplatin as measured by MTT assays.Kaplan–Meier plots of the survivals of CKIε-positive and CKIε-negative tumours.Univariate and multivariate analyses of the CKIε positivity together with other clinicopathological parameters in predicting clinical outcome.

To study the effects of selective CKIε inhibition and chemotherapeutic agents on ovarian cancer cells, we compared ovarian cancer cells which had normal expression of CKIε (control shRNA) to ovarian cancer cells which had suppressed CKIε expression (CKIε shRNA). Doses of carboplatin and paclitaxel were added separately to the cell lines and up-titrated in a standard fashion. Compared to control shRNA cells, CKIε shRNA cells had a more significant response to escalating doses of carboplatin and paclitaxel (Fig 5A). To demonstrate that reduced level of ANT2 is responsible for the enhanced sensitivity of the cells to chemotherapeutic agents, ANT2 levels in the wild-type cancer cells were suppressed by two small interfering RNAs (siRNAs) (Supporting Information Fig S10B). Cancer cells receiving the ANT2 siRNAs were significantly more susceptible to carboplatin and paclitaxel treatments than the cancer cells receiving control siRNA (Supporting Information Fig S10C). To test the effects of ANT2 suppression in a CKIε-overexpressing context, we transfected anti-ANT2 siRNA1 to control HOSE cells and CKIε-overexpressing HOSE cell line 3. The CKIε-overexpressing cells showed a more pronounced 26% decrease in growth when compared to control HOSE cells, which had a decrease of 11.5% (Supporting Information Fig S10D). Together, these findings demonstrate that ANT2 mediates the effects of CKIε on cell growth and CKIε-selective inhibition reduces ANT2 level in mitochondria and renders ovarian cancer cells more susceptible to chemotherapeutic agents.

### Ovarian cancer tumours which overexpress CKIε have a worse prognosis

To extend our findings to the clinical setting, we analysed the CKIε immunohistochemical staining data of the 57 invasive ovarian carcinomas that have survival information together with other clinicopathological parameters. By using the median CKIε expression level of 3 as cut-off in the invasive tumours, we found that patients with a score ≤3 had a mean overall survival (OS) of 101 months. In contrast, patients with a score >3 had a mean OS of 59 months (Fig 5B). A multivariate analysis (Fig 5C) of the data also found that positive CKIε expression, besides stage, is an independent predictor of survival (Hazard ratio is 2.652, 95% CI = 1.10–6.39, *p* = 0.03). Further Kaplan–Meier estimation of survival functions indicated that serous tumours with CKIε-positivity has worse survival (mean survival = 45.999 months, 95% CI = 17.0–75.0) compared to CKIε-negative tumours (mean survival = 107.471 months, 95% CI = 52.9–162.1) with a *p*-value of 0.036 (Tables S1 and S2). These findings strongly suggest that patients whose tumours overexpress CKIε are associated with a worse prognosis than patients whose tumour does not express CKIε.

## DISCUSSION

CKIε has been shown to be important in several biologic activities including the circadian clockwork (Lee et al, 2009), developmental polarity (Tsai et al, 2007) and in phosphorylating key proteins in cancer signalling pathways such as p53 and β-catenin (Gao et al, 2002; Meng et al, 2010; Seifert & Mlodzik, 2007; Yang & Stockwell, 2008). However, the significance of CKIε in ovarian cancer has not been investigated and its potential as a therapeutic target has not been explored. By inducing ectopic expression of CKIε in normal HOSE cells, we were able to demonstrate an accelerated cellular proliferation rate as well as an enhanced ability of ectopic CKIε-expressing cells to form large 3D spheroids. Conversely, growth rates were significantly reduced by adding pharmacologic inhibitors of CKIε/δ (PF-670462 and PF-4800567) to ovarian cancer cell lines. Moreover, cancer cells with suppression of CKIε expression using shRNA constructs that targeted the CKIε gene had reduced growth rates, a reduced capacity to migrate and reduced tumour growth *in vivo* (Fig 3). As our detailed analyses have shown that the closely related CKIδ was sparsely expressed in ovarian epithelial cells and the CKIα isoform is also highly expressed in normal ovarian surface epithelial cells, our findings strongly suggest that overexpression of CKIε isoform is responsible for cancer cell growth and migration.

The role of CKIε in the canonical pathway is the phosphorylation of Dsh/Dvl for the stabilization of β-catenin (Gao et al, 2002; Kishida et al, 2001). However, ovarian cancer cells have significant overexpression of β-catenin, which does not seem to be regulated by CKIε, as we did not find changes in either the level and activity of β-catenin in the knockdown cell lines. Characterization of mouse xenograft tumours with knockdown of CKIε also showed no changes in the β-catenin pathway components compared with xenograft tumours with wild-type cancer cells (Supporting Information Fig S6C). Our attempt to characterize the pathway by which CKIε elicited its effects led us to uncover the distribution of CKIε in mitochondria and its novel interaction with mitochondrial proteins Annexin A2, Prohibitin and ANT2. These novel interactions have been validated by reciprocal co-immunoprecipitation, confocal immunofluorescence co-localization experiments and Western blot analysis of proteins in the mitochondrial fraction. It has been reported that Annexin A2 can form high-molecular weight complexes with Prohibitin in the mitochondria (Bacher et al, 2002), which may constitute a chaperon function for ANT2 and CKIε interaction (Barath et al, 1999; Jang et al, 2008). The distribution of CKIε in mitochondria and its interactions with mitochondrial proteins suggest that CKIε might have a regulatory function in mitochondria independent of β-catenin. It is also noted that there were significant levels of both CKIε and ANT2 in the nuclear fraction (Supporting Information Fig S9), which might be caused by impurities derived from the membrane fractions.

In addition to showing suppressed levels of CKIε, CKIε knockdown ovarian cancer cells also revealed decreased levels of ANT2 *in vitro* and *in vivo*. ANT2 is overexpressed in highly proliferating cancer cells (Barath et al, 1999; Jang et al, 2008) and is involved in the maintenance of mitochondrial membrane potential, energy homeostasis, tumour growth and resistance to chemotherapy-induced apoptosis (Jang et al, 2008; Le Bras et al, 2006). Our determination of ATP content in control shRNA cells and in CKIε shRNA cells showed significantly reduced ATP levels in CKIε shRNA cells. Prior reports have indicated that inhibition of ANT2 resulted in apoptosis and in chemosensitization (Le Bras et al, 2006). Indeed, the CKIε knockdown cells had a more robust response to the chemotherapeutic agents carboplatin and paclitaxel when administered individually. We have also shown that ANT2 is overexpressed in ovarian tumours and suppression of ANT2 expression through siRNAs reduced cell growth in CKIε-overexpressing HOSE cells and sensitized ovarian cancer cells to chemotherapeutic agents. This data strongly suggests that the impact of CKIε on cancer cell survival and chemotherapeutic response is mediated through CKIε complex formation in the mitochondria and ANT2 regulation. Besides ANT2, other complex components such as Prohibitin may also contribute to drug resistance, as suggested in a recent report (Patel et al, 2010). Our clinical data indicate that patients whose tumour over-expresses CKIε had a worse prognosis than patients whose tumour did not over-express CKIε are also consistent with the protective function of CKIε on tumour cell growth and chemoresponse.

In conclusion, our studies have uncovered a novel mitochondrial pathway for CKIε, which regulates the expression of metabolic and apoptosis-regulating protein ANT2. The expression of ANT2 was related to CKIε status and cellular level of CKIε is negatively related to cellular chemosensitivity and clinical response. Hence, the cellular function of CKIε might be mediated through the downstream target ANT2 in the mitochondria. Furthermore, our data add ovarian cancer to the growing list of malignant tumours that shows upregulation of CKIε expression in cancer compared to control normal tissues (Brockschmidt et al, 2008; Firestein et al, 2008; Frierson et al, 2002; Kim et al, 2010; Yang & Stockwell, 2008). The results of our study and the availability of small molecule inhibitors of CKIε may be of particular importance in patients with ovarian cancer whose tumour over-expresses CKIε. Although pharmacologic inhibition of CKIε using PF-670462 and PF-4800567 alone did not result in drastic cell death, the involvement of CKIε in regulating an important factor in mitochondrial function presents the potential of targeting CKIε in enhancing response rates to chemotherapy. There is ample evidence to support the efficacy of mitochondrially targeted agents to cooperate with conventional chemotherapy and radiation to eradicate chemotherapy-refractory cancer cells (Fulda et al, 2010; Galluzzi et al, 2006; Kang & Reynolds, 2009). The response of our CKIε knockdown cell lines to standard ovarian cancer chemotherapeutic agents further supports this notion. In the last 20 years, survival rates in patients with advanced ovarian cancer have stagnated. Our data provides substantial evidence that CKIε harbours tumourigenic properties and is critical to cellular proliferation and migration in ovarian cancer cells. Inhibiting CKIε resulted in decreased cell growth rates and tumour burden, and acted as a potent sensitizer to chemotherapeutic agents. Results from prior studies as well as the data in this report provide compelling evidence for pursuing CKIε as a therapeutic target in future clinical trials.

## MATERIALS AND METHODS

### Ovarian clinical samples and ovarian cell lines

All patient-derived biologic specimens were collected and archived under protocols approved by the Human Subjects Committee of the Brigham and Women's Hospital, Boston, Massachusetts. Clinical samples were collected with written informed consent from patients and confirmed histologically by gynaecologic pathologists. Cases were staged according to International Federation of Gynecology and Obstetrics (FIGO) system. The normal HOSE cells and ovarian cancer cell lines have been described previously (Huang et al, 2006). All ovarian cell lines were maintained in a mixture of medium 199 and MCDB105 medium (1:1) (Sigma, St. Louis, MO) supplemented with 10% foetal calf serum (Invitrogen, Carlsbad, CA).

### Immunohistochemistry

Expression of CKIε was studied in 76 ovarian tumours by IHC. Deparaffinization of paraffin embedded ovarian tissue sections was performed using xylene and rehydration with a graded ethanol series. Antigen retrieval was performed in a pressure-cooker in antigen-unmasking solution (Vector Laboratories, Burlingame, CA) for 10 min. The reaction was visualized using a horseradish peroxidase-based Vectastain Elite ABC Kit with diaminobenzidine chromogen as a substrate (Vector Laboratories). Primary antibodies used included monoclonal mouse anti-human CKIε (1:70 dilution; BD Biosciences, San Jose, CA), rabbit anti-human CKIε antibody and blocking peptides (Abgent, San Diego, CA), rabbit anti-human CKIα antibody (LifeSpan Biosciences, Seattle, WA), goat anti-human ANT2 (1:100 dilution, Santa Cruz Biotech, Santa Cruz, CA) and anti-CKIδ antibody (Abcam, Cambridge, MA). Two trained observers scored the slides independently, and the scores for all cases were compared for discrepancies.

### Genomic DNA amplification and sequencing

The primer sequences for amplifying 5′-genomic coding fragment of CKIε were: 5′-CCATCCTCTGGCATCCTCT-3′ and 5′-CACACGCCAGATCTCAGAAA-3′. Genomic DNA was amplified using Phusion® High-Fidelity DNA Polymerase (New England Biolabs, Ipswich, MA) according to the manufacturer's instruction. Amplified DNA was sequenced using sequencing primer 5′-GACTGCCTGGCCTTTGAG-3′ at Harvard Medical School DNA Resource Core. Sequence chromatograms were visually reviewed and the sequences were also aligned to the wild-type sequence at Human RefSeq Genomic dataset using NCBI BLAST® program (http://blast.ncbi.nlm.nih.gov/) for any potential genetic mutations.

**The paper explained**

PROBLEM:

Epithelial ovarian cancer is the most lethal gynaecologic malignancy in Western countries (Jemal et al, 2010). Over 70% of ovarian cancer cases are diagnosed in the advanced stage of the disease, which confers an overall survival of 30% at 5 years (Bast et al, 2002; Kosary, 2008). Standard treatment for advanced ovarian cancer consists of cytoreductive surgery followed by platinum-based chemotherapy and a taxane (McGuire et al, 1996; Ozols et al, 2003). However, chemotherapy related toxicities and drug resistant tumours are significant barriers to treatment. Recent genomic analyses of many human cancers have revealed that a significant number of tumours have alterations in a few core pathways (Luo et al, 2003; McCormick, 1999; Rodriguez-Viciana et al, 2004; Wullschleger et al, 2006). Identifying and characterizing these core pathways provides a foundation for therapeutic development. Here we report the identification of casein kinase I-epsilon (CKIε), a Wnt signalling protein that is overexpressed in ovarian tumours. Our research has shown that this protein is critical in answering our question: How to target chemoresistance of ovarian cancer cells?

RESULT:

Employing a variety of biochemical and immunotechnical assays including cell fractionation and immunoprecipitation assay, we are able to demonstrate that the functional importance of CKIε is related to its regulation of expression of a mitochondrial protein, adenine nucleotide translocase 2 (ANT2). Inhibition of CKIε suppressed the expression of ANT2 and lowered intracellular ATP level, which was associated with hypersensitivity of resulting cells to therapeutic agents. In support of this finding, clinical ovarian cancer cases with high levels of CKIε consistently were associated with poor outcome.

IMPACT:

Emergence of drug resistance to standard chemotherapy presents a serious challenge to overall survival of ovarian cancer patients. New strategy that targets key survival mechanism such as CKIε may have a high impact on future prognosis of ovarian cancer patients.

### Establishment and characterization of cell lines with changes of CKIε expression

Mission™ lentiviral CKIε-targeting and non-target control shRNA transduction particles and Mission siRNAs were purchased from Sigma–Aldrich (St. Louis, MO). Infection of MCAS and SKOV3-IP^Luc^ ovarian cancer cells with lentiviral transduction particles and puromycin selection was performed according to the manufacturer's instruction. Knockdown of CKIε expression in the resultant cell lines was confirmed by Western blot analysis. For the introduction of CKIε into HOSE cells, full-length CKIε cDNA expression construct and empty vector were purchased from OriGene Technologies (Rockville, MD). Transfection of CKIε cDNA and ANT2-targeting siRNAs was performed using Lipofectamine™ 2000 transfection reagent (Invitrogen Corp. Carlsbad, CA). Characterization of the resulted cell lines was performed as described in Supporting Information Materials and methods.

### Mouse xenografts

Animal protocol was reviewed by Standing Committee on Animals at Harvard Medical School. For mouse xenografts, 5 × 10^6^ SKOV3-IP^Luc^ cells harbouring control shRNA or shRNA construct 3 were injected into the peritoneal cavity of 12 athymic female nude mice (Taconic, Hudson, NY). After 28 days, mice were administered intraperitoneally with d-luciferin (150 mg/kg body weight) and whole-body bioluminescent images were taken and analysed using a Xenogen IVIS-Imaging System 100 Series (Caliper LifeSciences, Hopkinton, MA). The mice were sacrificed and the tumours were harvested and weighed and further characterized by IHC and Western blot analysis.

### Co-immunoprecipitation and protein identification

Standard Co-immunoprecipitation assays were performed as described in Supporting Information Materials and methods. After overnight incubation of the lysates with the antibody of interest, the immune complex was captured by protein A/G immobilized on agarose beads (Pierce Biotechnology, Rockford, IL), fractionated by standard SDS-PAGE, transferred to PVDF membrane (Pierce Biotechnology) and analysed by Western blot. To identify unknown proteins, the gel was stained using the sensitive Colloidal Coomassie universal protein dye (Invitrogen Corp.). The band for the protein of interest was excised from the gel and sent to the Taplin Biological Mass Spectrometry Facility at Harvard Medical School for protein identification by mass spectrometry.

### Immunofluorescence microscopy

Wild-type MCAS cells were fixed in 4% paraformaldehyde (Sigma–Aldrich) and permeabilized with PBS containing 0.5% Triton X-100 (Sigma–Aldrich). After blocking with 10% foetal bovine serum (Invitrogen), primary antibodies were added and incubated at 24°C for 2 h. Anti-mouse antibody coupled with Alexa Fluor 647 and Alexa Fluor 546-conjugated anti-rabbit and anti-goat secondary antibodies (Invitrogen) were used to stain CKIε, and mitochondrial proteins, respectively. The stained cells were counterstained with Sytox Green (Invitrogen). Microscopic images were captured by Leica SP5 confocal microscope (Leica Microsystems, Bannockburn, IL) and analysed by the Leica LAS AF software (Leica Microsystems).

### Subcellular protein fractionation and Western blot analysis

Nuclear and cytosolic fractions were isolated according to Ng et al (1995). Mitochondria were isolated according to Frezza et al (2007). Extracted proteins were resuspended in RIPA lysis buffer (50 mM Tris HCl pH 8, 150 mM NaCl, 1% NP-40, 0.5% sodium deoxycholate and 0.1% SDS) supplemented with PhosStop phosphatase inhibitor cocktail and complete protease inhibitor cocktail (Roche Applied Science, Indianapolis, IN) and protein concentration was measured with a MicroBCA protein assay kit (ThermoScientific, Rockford, IL). Standard sodium dodecyl sulfate-polyacrylamide gel electrophoresis (SDS-PAGE) and Western blot analysis using a Supersignal west pico kit (Pierce Biotechnology) was performed as described in Supporting Information Materials and methods.

### Statistical analysis

All calculations were performed with MINITAB statistical software (Minitab, State College, PA) unless otherwise indicated. ANOVA was used to compare the mean IHC scores between benign and malignant paraffin sections and between different tumour histologies. Significance of the test was considered at the 5% level (*i.e. p*-value ≤ 0.05). Overall survivals of patients with positive and negative CKIε expression were plotted using the Kaplan–Meier method, and compared with a log rank test. The impact of CKIε expression on patient survival was further studied with the inclusion of potential clinical risk factors using the Cox proportional hazards regression model and analysed using a forward stepwise Wald-test process. The Cox regression analysis was performed using the SPSS 17.0 statistical software. (SPSS, Inc., Chicago, IL).

For more detailed Materials and Methods see the Supporting Information.

## For more information

For SEER Cancer Statistics:: http://seer.cancer.gov/statistics/

For more information regarding ovarian cancer:

American Cancer Society: http://www.cancer.org/cancer/ovariancancer/

OvarianCancerPrognosis.net: http://www.ovariancancerprognosis.net/

Ovarian Cancer Research Fund: http://www.ocrf.org/

The Marsha Rivkin Center for Ovarian Cancer Research: http://www.marsharivkin.org/

OMIM site for Casein Kinase I epsilon:: http://omim.org/entry/600863

Adenine Nucleotide Translocator 2:: http://omim.org/entry/300150

## Supplementary material

Detailed facts of importance to specialist readers are published as ”Supporting Information”. Such documents are peer-reviewed, but not copy-edited or typeset. They are made available as submitted by the authors.
