# Supplementary material for: Casein kinase I epsilon interacts with mitochondrial proteins for the growth and survival of human ovarian cancer cells
Source: EMBO Mol Med. 2012 Jun 18;4(9):952–63. doi: 10.1002/emmm.201101094 (PMC3491827; doi:10.1002/emmm.201101094)
Supplement: Supplementary file 1 [file emmm0004-0952-SD1.pdf]

EMBO
Molecular Medicine

Manuscript EMM-2011-01094

CASEIN KINASE I EPSILON INTERACTS WITH MITOCHONDRIAL PROTEINS FOR THE GROWTH AND SURVIVAL OF HUMAN OVARIAN CANCER CELLS

Noah Rodriguez, Junzheng Yang, Kathleen Hasselblatt, Shubai Liu, Yilan Zhou, Jose A. Rauh-Hain, Shu-Kay Ng, Pui-Wah Choi, Wing-Ping Fong, Nathalie Y.R. Agar, William R. Welch, Ross S. Berkowitz and Shu-Wing Ng

Corresponding author: Shu-Wing Ng, Brigham and Women's Hospital

Review timeline:

Submission date:	23 November 2011
Editorial Decision:	21 December 2011
Revision received:	02 April 2012
Editorial Decision:	30 April 2012
Revision received:	07 May 2012
Accepted:	11 May 2012

Transaction Report:

(Note: With the exception of the correction of typographical or spelling errors that could be a source of ambiguity, letters and reports are not edited. The original formatting of letters and referee reports may not be reflected in this compilation.)

1st Editorial Decision 21 December 2011

Thank you for the submission of your manuscript to EMBO Molecular Medicine. We have now heard back from the three referees whom we asked to evaluate your manuscript.

As you will see, although they find your study of potential interest, they also raise a number of concerns about the conclusiveness of the results and several technical issues. Referee 1 feels that clinical relevance should be improved by further analyzing i) the xenografted tumors and ii) patient's survival in relation to CK1e expression and type of malignancy. Referee 3 however, considers that the study is too limited on a mechanistic level to envisage revision. Nevertheless, referees 1 and 2 have suggested that the manuscript might be published in EMBO Molecular Medicine after a major revision.

Given that all of them find the message of the study novel and interesting we would be willing to consider a revised manuscript with the understanding that the referees' concerns must be fully addressed and that acceptance of the manuscript would entail a second round of review.

I should remind you that it is EMBO Molecular Medicine policy to allow a single round of revision only and that, therefore, acceptance or rejection of the manuscript will depend on the completeness of your responses included in the next, final version of the manuscript. I realize that addressing the referees' comments in full would involve a lot of additional experimental work and I am uncertain whether you will be able (or willing) to return a revised manuscript within the 3 months deadline and I would also understand your decision if you choose to rather seek rapid publication elsewhere at this stage.

I look forward to seeing a revised form of your manuscript as soon as possible.

Should you find that the requested revisions are not feasible within the constraints outlined here and choose, therefore, to submit your paper elsewhere, we would welcome a message to this effect.

Yours sincerely,

Editor
EMBO Molecular Medicine

***** Reviewer's comments *****

Referee #1 (Comments on Novelty/Model System):

1. The experiments are carefully performed. In some cases adaquate controls are missing.

2. Novelty:
The manuscript includes interesting and novel data, especially the interaction between CK1e and ATN2 which has not described before.

3.Understanding the role of CK1 isoforms in tumorigenesis is of general interest. Furthermore, CK1e is a new therapeutic drug target and inhibition of CK1e seems to increase sentitivity towards chemotherapeutics.

4. The experimental setups are suitable to analyze the different questions.

Referee #1 (Other Remarks):

In this paper Rodriguez and co-workers show that CK1e interacts with mitochondrial proteins and that the expression level of CK1e correlates with the survival of patients. Although the manuscript includes interesting and novel data, the manuscript needs accuracy in citing and additional experimentation to enhance the scientific stringency.
The following points are listed in the order in which they appear in the paper:

1. Figure 1A: The specificity of the CK1 epsilon staining in paraffin embedded specimen should be improved by
(i) performing IHC in the presence of increasing amounts of either unspecific or CK1e specific peptides,
(ii) using a second CK1e specific antibody, and
(iii) staining of CK1e in established ovarian tumor cells which have been fixed and embedded in paraffin under the same conditions as the tissue specimens.

2. Figure 1C:
2a) The expression levels of CK1e in all analyzed cell lines should be quantified.
2b) Are there differences in the CK1e specific kinase activity detectable in the analyzed cell lines?
2c) Do all analyzed cell lines express wild-type CK1e, or are there mutations within the coding regions of CK1e variant 1 and variant 2?
2d) Furthermore, the expression levels of CKd and CK1a should be determined by Western Blot analyses, quantified and compared with the expression levels of CK1e.

3. Figure 2B: Are there differences in the subcellular localization, the expression and/or activity levels of CK1e in cells grown as monolayers compared to those grown as spheroids?

4. The authors analyzed the effects of two CK1 specific inhibitors in six ovarian cancer cell lines. The authors should clearly state in the text and figure legend of figure 3 that they observed a reduction of cell viability in the presence of both inhibitors. However, the authors only observed a

50% reduction in cell viability at inhibitor concentrations which are much higher than the published IC50 values. Therefore, off-target effects cannot be excluded. In addition, since both inhibitors inhibit CK1d and e to a similar extend (IC261; IC50: CK1d/e 1 uM, Mashhoon et al., 2000; PF-670462, IC50 CK1d 14 nM, CK1e 7 nM, Badura et al., 2007) the conclusion drawn by the authors is not conclusive.
4a) Therefore, additional inhibitors differing more significantly in their IC50 values for CK1d and CK1e should be used, e.g. PF-4800567 (Walton et al., 2009), 1-hydroxy-4-amino-anthraquinone (Cozza et al., 2009), or imidazole compound 18 (Peifer et al., 2009). Furthermore, the authors have to consider that not all effects of IC261 are mediated by inhibition of CK1d/e (Cheong et al., 2011). Whereas the use of IC261 in biochemical assays still leads to clear results, it is difficult to decide whether the cellular effects upon IC261 treatment are mediated by inhibition of CK1d/e or by affecting microtubule stability (Cheong et al., 2011, Iszeradjene et al., 2004).
4b) To further analyze the effects of pharmacological inhibition of CK1e, the effects of CK1 specific inhibitors on cell cycle distribution should be analyzed additionally.

5. The authors clearly show that the knock-down of CK1e suppresses cell proliferation. To underline the specifity of the used CK1e specific shRNAs western blot analysis for CK1d should be shown.

6. Furthermore, it might be possible that suppression of CK1d also lead to an inhibition of cell proliferation. Therefore, the authors should analyze the effects of CK1d specific shRNA on proliferation of ovarian tumor cell lines.

7. The data provided indicate that upon injection of SKOK3-IPLuc - CK1e shRNA cells the induced tumor growth in xenografts was significantly lower compared to injection of control cells. However, no further analyses of the tumors are shown. Did the authors observed differences in the grading and staging of the tumors? In addition, a comparison of their proliferation rate (Ki-67 staining) and apoptotic rate (e.g. TUNEL assay) is missing. Furthermore, comparative analyses regarding changes in signal transduction pathways should be performed (e.g. microarrays and/or quantitative real-time PCR analyses).

8. The authors show that high CK1e expression is associated with a worse prognosis of the patients. Considering that the different histological subgroups differ in their malignancy, the effects of CK1e expression levels on the survival of the patients should be analyzed in all histological subgroups separately.

9. Mutations within the coding region of CK1e have been detected in mammary tumors. These mutations lead to an increase of the oncogenic potential of CK1e. Therefore, the authors should also analyze the occurrence of mutations within the coding regions of both CK1e variants in ovarian tumors.

10. Discussion: Several relevant publications are not cited and discussed (Fuja et al., 2004, Dolezal et al., 2010, Foldynova et al. 2010), or are not exactly interpretated (e.g. Behrend et al., 2000).

Referee #2 (Comments on Novelty/Model System):

This is potentially an important paper. The relationship between tumor phenotype and expression of casein kinase 1 epsilon in ovarian cancer has not been reported previously. This accounts for the highly novel aspect of the work. Technical aspects, such as evaluating expression using immunohistochemistry, although adequate, are not always ideal. More experiments will need to be done on this system before it could be considered to have a likely medical impact. The same is true for any other early research project on gene expression and tumor phenotype.

Referee #2 (Other Remarks):

The manuscript describes a study of the biological effects of casein kinase I-epsilon (CKI), which the authors found to be frequently overexpressed in epithelial ovarian carcinoma. Using forced overexpression of this gene and inhibition using shRNAs they found that CKIe has a role in cellular proliferation and migration. They also found that the CKIe expression had no effect on beta-catenin

activity, but seemed to cause a decrease in expression of ANT2 and a consequent increase in sensitivity to several chemotherapeutic agents. Overall, the paper was interesting and thought provoking. I have several suggestions to improve the presentation.

1. For the table in figure 1B, it would be better to describe the data with more than just a mean score for each group. Results for each patient are likely different with some having high and some low expression of CKIe. This can be presented using a scatter plot (for example Figure 2 in Nature Genetics, 2009, vol. 41, p. 1100). The results for patients and controls should be presented this way.
2. In figure 1C, the names of the cell lines should be given.
3. The brackets and P<0.05 above the brackets in the two graphs in figure 3A imply that the statistical evaluation is a comparison of two different concentrations of the CKIe inhibitors. I don't think that is what was intended for the statistical evaluation. Please clarify, both in the text and the figure what is being evaluated by the statistics.
4. The legend for figure 3D, right side, should describe the measures of dispersion used in the graph.
5. For the experiments described in figure 3D the CKIe shRNA group had much less tumor growth. For the tumors that did eventually grow, was the shRNA still being expressed and CKIe still being suppressed? If this tumor material was saved it may be possible to do this experiment.
6. From the perspective of therapy, it would be useful if the authors could try the CKIe inhibitors used in figure 3A in an experiment similar to that in figure 5C.

Referee #3 (Comments on Novelty/Model System):

The manuscript submitted by Rodriguez et al., describes the identification of a novel interaction between CASEIN KINASE I EPSILON (CKI) and ANT2, a mitochondrial protein involved in the exchange of ADP and ATP across the inner membrane and in some cancers. The manuscript is well written, interesting and findings are original. However, to my opinion results regarding the molecular interplay between both proteins do not support the interpretation raised by the authors. Moreover, the choice of experiments was not the most pertinent to address the biological question.

Referee #3 (Other Remarks):

Despite a potential strong impact of the paper in the field of cancer prognosis and therapeutics, I feel that the first part of the manuscript reaches the quality to be published in a high standard journal, the second part, which concerns the molecular mechanisms is clearly inadequate. As a result, I think that too much experiments are needed to ask a revision of the paper.

In the manuscript, the authors suggest that CKI is involved in epithelial ovarian carcinoma. The data regarding the role of CKI in growth and survival of human ovarian cancer cells are convincing. However, the mechanistic part of the manuscript is not convincing enough in my opinion. The authors claim that CKI interacts with mitochondrial proteins (ANT2, Annexin A2 and Prohibitin) and that this interaction might play a role in the proliferation of ovarian cancer cells. Unfortunately, the data provided does not support this statement.
First of all, the interaction of the four proteins is not proved: Figure 4B shows that CKI interacts with each of the 3 mitochondrial proteins and Figure S5 shows that each protein can interact with CKI separately. The author should show the presence of the four proteins in each co-immunoprecipitation. Moreover, ANT2 expression is dependent on CKI expression as shown in Figure 4D, which is not the case for Annexin A2 or Prohibitin, suggesting a different mechanism for these proteins. Next, the authors argue that ANT2 is responsible for the susceptibility of ovarian cancer cells to chemotherapeutic agents. But the data in figure 5 only show a similar effect of siRNA directed against ANT2 and shRNA directed against CKI . To prove this, the authors should perform overexpression of ANT2 in their shRNA CKI cells and show a recovery of the proliferation of the cells or perform silencing of ANT2 in CKI overexpressing cells and show that the proliferative effect of CKI is suppressed by ANT2 depletion.
Finally, CKI is a kinase and the authors should assess the phosphorylated state of the mitochondrial partners of CKI as a possible regulatory mechanism.

1st Revision - Authors' Response 02 April 2012

We are very pleased that all of the reviewers found the message of our study novel and interesting and your consideration of publishing a revised manuscript. We have performed additional experiments as requested by the reviewers and made a major revision of the manuscript. We are addressing the reviewers' comments and suggestions as follows:

Referee #1:

Although the manuscript includes interesting and novel data, the manuscript needs accuracy in citing and additional experimentation to enhance the scientific stringency.

The following points are listed in the order in which they appear in the paper:

1. Figure 1A: The specificity of the CK1 epsilon staining in paraffin embedded specimen should be improved by

(i) performing IHC in the presence of increasing amounts of either unspecific or CK1e specific peptides:

We have performed IHC in the presence of blocking peptides for CKIe and the signal was significantly diminished, suggesting the specificity of the staining.

(ii) using a second CK1e specific antibody:

We have also performed IHC using another CKIe-specific antibody from Abgent. The results of the second antibody were the same as the first one.

(iii) staining of CK1e in established ovarian tumour cells which have been fixed and embedded in paraffin under the same conditions as the tissue specimens.

We have also stained the CKIe expression in ovarian cancer cells prepared using the fixation and paraffin embedding procedure as for the tissue specimens. The staining showed similar intensity as in the tumour tissues.

All the results were presented in Supplemental Figure 1.

2. Figure 1C:

2a) The expression levels of CK1e in all analyzed cell lines should be quantified.

We have quantified the expression levels relative to beta-actin levels and added to Figure 1C.

2b) Are there differences in the CK1e specific kinase activity detectable in the analyzed cell lines?

We could not perform kinase assays because we needed a valid permit to use radioisotopes. However, we have examined the phosphorylation status of CKIe-co-immunoprecipitated products in the cell lines and confirmed phosphorylation of ANT2 and/or Prohibitin, which could be resulted by the kinase activity of CKIe. The result is shown in Figure S8B.

2c) Do all analyzed cell lines express wild-type CK1e, or are there mutations within the coding regions of CK1e variant 1 and variant 2?

Fuja *et al. have reported somatic mutations of CK1e in mammary ductal carcinoma. We have designed primers to PCR the proposed 5'-coding region of the gene from microdissected DNA prepared from a panel of 38 ovarian carcinomas and genomic DNA from 8 ovarian cancer cell lines. The results showed that no mutations were identified from the PCR products. The results*

are shown in Supplemental Figure S3.

2d) Furthermore, the expression levels of CKd and CK1a should be determined by Western Blot analyses, quantified and compared with the expression levels of CK1e.

We have compared the levels of CKId and Ck1a with CK1e in the ovarian cell lines by Western blot analysis. In ovarian epithelial cells, we did not find significant expression of CK1d in the ovarian cell lines. There are no significant differences in CKIa expression between normal and cancerous ovarian epithelial cells. Hence, only the CKIe isoform shows differential expression between normal and cancerous ovarian epithelial cells. The Western blot analysis results are shown in Figure S2.

3. Figure 2B: Are there differences in the subcellular localization, the expression and/or activity levels of CK1e in cells grown as monolayers compared to those grown as spheroids?

We have stained the CKIe-overexpressing HOSE cells growing as spheroids. The CKIe staining was located in the cytoplasm of the cells lining the lumens of the spheroids. The result is shown in Supplemental Figure S4A.

4. The authors analyzed the effects of two CK1 specific inhibitors in six ovarian cancer cell lines. The authors should clearly state in the text and figure legend of figure 3 that they observed a reduction of cell viability in the presence of both inhibitors. However, the authors only observed a 50% reduction in cell viability at inhibitor concentrations which are much higher than the published IC50 values. Therefore, off-target effects cannot be excluded. In addition, since both inhibitors inhibit CK1d and e to a similar extend (IC261; IC50: CK1d/e 1 uM, Mashhoon et al., 2000; PF-670462, IC50 CK1d 14 nM, CK1e 7 nM, Badura et al., 2007) the conclusion drawn by the authors is not conclusive.

4a) Therefore, additional inhibitors differing more significantly in their IC50 values for CK1d and CK1e should be used, e.g. PF-4800567 (Walton et al., 2009), 1-hydroxy-4-amino-anthraquinone (Cozza et al., 2009), or imidazole compound 18 (Peifer et al., 2009). Furthermore, the authors have to consider that not all effects of IC261 are mediated by inhibition of CK1d/e (Cheong et al., 2011). Whereas the use of IC261 in biochemical assays still leads to clear results, it is difficult to decide whether the cellular effects upon IC261 treatment are mediated by inhibition of CK1d/e or by affecting microtubule stability (Cheong et al., 2011, Iszeradjene et al., 2004).

As most ovarian epithelial cells do not express Ck1d, the effects of the drugs on Ck1d is minimal at best. We have performed MTT assay with a highly CK1e-targeting inhibitor, PF-4800567. The result (added to Figure 3A) was similar to that of PF-670462. After more characterization experiments, we agreed with the reviewer that the cellular effects of IC261were not only due to CKIe inhibition and we focused on the other two inhibitors.

4b) To further analyze the effects of pharmacological inhibition of CK1e, the effects of CK1 specific inhibitors on cell cycle distribution should be analyzed additionally.

We have analyzed the cancer cells after inhibitors treatment by flow cytometry. The treatment of cells with IC261 showed significant arrest of cell cycle at G2/M, confirming the effects of the drug on microtubule stability as reported. However, both PF-670462 and PF-4800567 showed an increase of cells in G1 phase. The results of cell cycle distribution are presented in Supplemental Figure S5.

5. The authors clearly show that the knock-down of CK1e suppresses cell proliferation. To underline the specificity of the used CK1e specific shRNAs western blot analysis for CK1d should be shown.

We have shown by Western blot (Figure S2) that most of the ovarian epithelial cell lines did not express CK1d. Hence, the shRNAs should specifically target only CKIe.

6. Furthermore, it might be possible that suppression of CK1d also lead to an inhibition of cell proliferation. Therefore, the authors should analyze the effects of CK1d specific shRNA on proliferation of ovarian tumour cell lines.

   Since ovarian epithelial cells do not express CK1d, we have not performed a CKId knockdown experiment.

7. The data provided indicate that upon injection of SKOK3-IPLuc - CK1e shRNA cells the induced tumour growth in xenografts was significantly lower compared to injection of control cells. However, no further analyses of the tumours are shown. Did the authors observed differences in the grading and staging of the tumours? In addition, a comparison of their proliferation rate (Ki-67 staining) and apoptotic rate (e.g. TUNEL assay) is missing. Furthermore, comparative analyses regarding changes in signal transduction pathways should be performed (e.g. microarrays and/or quantitative real-time PCR analyses).

   We have analyzed the xenograft tumours. Hematoxylin and eosin staining and staining for Ki-67 showed less robust growth of the knockdown tumours. No difference in apoptotic rate was observed. We have also stained the tissues and performed Western blot analysis for signaling pathway markers. There are no differences in nuclear b-catenin staining and Western blot analyses did not reveal any changes in b-catenin levels and phosphorylation of Dvl protein. The results are shown in Figure S6.

8. The authors show that high CK1e expression is associated with a worse prognosis of the patients. Considering that the different histological subgroups differ in their malignancy, the effects of CK1e expression levels on the survival of the patients should be analyzed in all histological subgroups separately.

   Because most of the 57 invasive tumours with survival data were high-grade serous tumours, we could not compare the CKIe effect for all histological subtypes because of small sample size. We used Kaplan-Meier estimation of survival functions to compare survival according to serous and non-serous tumour types (Tables S1 and S2). For the serous tumours, cases with CKIe positivity showed worse survival than cases with negative CKIe staining, with a significant P-value of 0.036. The non-serous cases did not show significance, probably due to small sample size.

9. Mutations within the coding region of CK1e have been detected in mammary tumours. These mutations lead to an increase of the oncogenic potential of CK1e. Therefore, the authors should also analyze the occurrence of mutations within the coding regions of both CK1e variants in ovarian tumours.

   We have sequenced the proposed 5'-genomic region of the CK1e gene that was reported to contain mutations. However, after sequencing of microdissected DNAs from 38 cases of tumour tissues and genomic DNA from 8 ovarian cancer cell lines, we did not identify any cited mutations. The results are shown in Figure S3.

10. Discussion: Several relevant publications are not cited and discussed (Fuja et al., 2004, Dolezal et al., 2010, Foldynova et al. 2010), or are not exactly interpreted (e.g. Behrend et al., 2000).

    We thank the suggestion by the referee #1 and have integrated the references into the text and discussed the information together with our findings and the potential significance related to ovarian pathogenesis.

Referee #2

Overall, the paper was interesting and thought provoking. I have several suggestions to improve the presentation.

1. For the table in figure 1B, it would be better to describe the data with more than just a mean score for each group. Results for each patient are likely different with some having high and some low expression of CKIe. This can be presented using a scatter plot (for example Figure 2 in Nature Genetics, 2009, vol. 41, p. 1100). The results for patients and controls should be presented this way.

   We have completed the scatter plots and presented them in Supplemental Figure S1A according to the advice by the reviewer #2.

2. In figure 1C, the names of the cell lines should be given.

   We have added the names of the cell lines in the Legends for Figure 1, as well as in the Figure S2.

3. The brackets and P<0.05 above the brackets in the two graphs in figure 3A imply that the statistical evaluation is a comparison of two different concentrations of the CKIe inhibitors. I don't think that is what was intended for the statistical evaluation. Please clarify, both in the text and the figure what is being evaluated by the statistics.

   We originally wanted to show the P-values for the differences between treated and untreated groups. We have according to the comments of the reviewer removed the brackets and explained in the legend of Figure 3A that the P values of differences between untreated and treated cells are all < 0.05 for certain drug doses.

4. The legend for figure 3D, right side, should describe the measures of dispersion used in the graph.

   We have added the information regarding dispersion to the legend for Figure 3D.

5. For the experiments described in figure 3D the CKIe shRNA group had much less tumour growth. For the tumours that did eventually grow, was the shRNA still being expressed and CKIe still being suppressed? If this tumour material was saved it may be possible to do this experiment.

   We have performed IHC and Western blot analysis with the xenograft tumours and the results showed that tumours from cancer cells harboring shRNA maintained very little CKIe expression compared with the tumours derived from control cancer cells. The results are shown in Supplemental Figure S6.

6. From the perspective of therapy, it would be useful if the authors could try the CKIe inhibitors used in figure 3A in an experiment similar to that in figure 5C.

   Unfortunately all the ovarian cancer cell lines in the lab were derived from high-grade and predominately the serous type of tumours. Most of the cell lines also overexpressed CKIe. It is difficult to get meaningful results from the small sample pool that are comparable with the results with tumour tissues.

Referee #3:

1. First of all, the interaction of the four proteins is not proved: Figure 4B shows that CKIe interacts with each of the 3 mitochondrial proteins and Figure S5 shows that each protein can interact with CKIe separately. The author should show the presence of the four proteins in each co-immunoprecipitation.

   We have repeated the IP experiments and showed that all four proteins were in the immune complex. The results are shown in Supplemental Figure S8.

2. ANT2 expression is dependent on CKIe; expression as shown in Figure 4D, which is not the case for Annexin A2 or Prohibitin, suggesting a different mechanism for these proteins.

   CKIe might affect the stability and hence the expression of ANT2 but not the other two proteins (Figure 4D and Figure S9). Even though their levels were not altered in the CKIe knockdown cells, they were in the same complex with CKIe and ANT2 and might serve a supportive role. It has been reported that Annexin A2 forms complexes with Prohibitin in the mitochondria, which might have a chaperone function (Bacher et al. Prohibitin and prohibitone are contained in high-molecular weight complexes and interact with alpha-actinin and annexin A2. Biochimie, 2002, 84:1207-1220). There is also a report that shows Prohibitin is involved in drug resistance (Patel et al., Rescue of paclitaxel sensitivity by repression of Prohibitin 1 in drug-resistant cancer cells, Proc Natl Acad Sci USA (2010) 107:2503-8).

3. The authors argue that ANT2 is responsible for the susceptibility of ovarian cancer cells to chemotherapeutic agents. But the data in figure 5 only show a similar effect of siRNA directed against ANT2 and shRNA directed against CKIe. To prove this, the authors should perform overexpression of ANT2 in their shRNA CKIe cells and show a recovery of the proliferation of the cells or perform silencing of ANT2 in CKIe overexpressing cells and show that the proliferative effect of CKIe; is suppressed by ANT2 depletion.

   The cancer cell lines we transfected with ANT2 siRNA already have overexpression of CKIe. To further prove that ANT2 mediates CKIe function using a cell line engineered with CKIe overexpression, we have transfected control siRNA and ANT2 siRNA to CKIe-overexpressing HOSE cells and control HOSE cells respectively and the results showed that ANT2 silencing slowed down the growth of CKIe-overexpressing cells, further supporting the notion that CKIe mediates its growth regulating effects through ANT2. The result is shown in Supplemental Figure S10D.

4. CKIe is a kinase and the authors should assess the phosphorylated state of the mitochondrial partners of CKIe as a possible regulatory mechanism.

   We have performed Western blot analyses using an antibody that was specific to phospho-threonine proteins to probe the CKIe immunoprecipitated products. The results in Supplemental Figure S8B showed that ANT2 and Prohibitin, but not Annexin A2, were phosphorylated in the immune complex.

We greatly appreciate the thoughtful and constructive comments of the three referees. We have thoroughly and clearly responded to all of their constructive comments by performing several additional experiments. We strongly feel that the revised manuscript is substantially strengthened by our responses to reviewers' comments. We hope that we will now receive a favorable decision regarding publication of the revised manuscript in the near future.

2nd Editorial Decision 30 April 2012

Thank you for the submission of your revised manuscript to EMBO Molecular Medicine. We have now received the enclosed reports from the referees that were asked to re-assess it. As you will see the reviewers are now globally supportive and I am pleased to inform you that we will be able to accept your manuscript pending the following final amendments:

As you can see below, Referee #1 is still concerned by a number of issues. I would particularly encourage you to rewrite and interprete more cautiously the results, as and where suggested. This referee also calls for additional data. While the suggested experiments would indeed strengthen the paper, at this stage I would encourage you to perform those only if you already have the necessary material (for IHC analysis of CKIa staining and regarding the phosphorylation data).

Please also indicate in all figure legends which primary antibody was used.

Another small concern is the quality of the Figures:
-please increase the size of the labels imbedded within the panels such as even if the figure is reduced, all labels will remain readable.
-make sure to leave some white space in between lanes not originating from the same western blot
-increase the figures resolution in general; texts and labels are not sharp when zoomed-in.

Please modify the references according to our guide to authors.

Please make sure that Figures S8 and S9 are complete and provide a Table of Content on the 1st page of the SI-single pdf file.

We would appreciate if you could submit your revised manuscript within two weeks. I look forward to seeing a revised form of your manuscript as soon as possible.

Yours sincerely,

Editor
EMBO Molecular Medicine

***** Reviewer's comments *****

Referee #1:

In the revised version Rodriguez and co-workers have addressed all issues which have been raised by the reviewers, although some questions are still not answered satisfactorily. Nevertheless the stringency of the manuscript has increased. At present the main weakness of the manuscript is the description and interpretation of the CK1 expression data and inhibitor experiments. Rewriting and more carefully interpretation of these results seems to be mandatory. Furthermore, the phosphorylation data are preliminary.

Analysis of the expression levels of CK1 isoforms: page 5: "While ovarian tumors stained positive for CK1e, ...weak to negative staining for CK1d, confirming that the positive staining comes from CKIe alone." I agree that the expression of CK1 isoforms differ in the tumors, but not that the staining comes from CK1e alone. Whereas in most cases low and high scores for CK1e expression was counted, the expression of CK1d was not detected or expressed very weakly. Furthermore, I would assume that CK1a is expressed differently in tumors presumably showing high expression levels in many tumors (therefore, showing IHC analysis for CK1a would increase the stringency of the paper). The author could state that CK1 isoforms are differently expressed in ovary tumors.

"CK1d, and the more distant CK1a expression in ovarian cell lines showed almost absence of CK1d expression and no significant overexpression of CK1a in the ovarian cancer cell lines".... Western blot results suggest that only the CK1e isoform is overexpressed...". The data presented in figure S2 clearly show, elevated expression of CK1d in one ovarian tumor cell line (Ovca 420). Furthermore, the expression levels of CK1a differ in the analyzed tumor cell lines. Whereas CK1a expression is down-regulated in 5 cases it is up-regulated in 3 cases (that means CK1e is not the only isoform up-regulated in ovarian tumor cell lines) (therefore, additional IHC analysis of CK1a expression in tumor samples might be of interest, see above).

The authors should also indicate in all figure legends which primary antibody had been used.

"Hence, it is apparent that over-expression of wild - type CK1e transcript occurs in ovarian cancer". Since the authors did not sequenced all exons of CK1e the interpretation of their sequence results have to be more carefully; they can¥t exclude the presence of mutations in those exons which have not been analyzed.

The CK1 inhibitor chapter (page 6/7) needs some rewriting and more carefully interpretation of the results. "Three pharmacological inhibitors of CK1e..." Note, all three inhibitors inhibit CK1

isoforms, some of them with higher affinity towards CK1d/e or CK1e. "Both treatments with PF670462 and PF-4800567 caused a slight increase in G1 phase...". The data presented in figure S5 are not convincing. Upon PF670462 treatment no G1 arrest is visible and also the effects of PF-4800567 are negligible. Furthermore, the cell cycle analyses for those two inhibitors are not in line with the results of the MTT assays. Furthermore, not all cell lines used for MTT analyses are mentioned in the legend of figure 3a (e.g. violet line in the PF670462 graph)

Finally the phosphorylation data presented are not convincing. First at all, the question 2b having been raised by referee 1 has not been answered. Instead a complete other aspect was addressed. The authors now wanted to know if the CK1e interacting mitrochondrial proteins (ANT2 and/or Prohibitin) are phosphorylated by CK1e. To solve this question additional experiments are necessary. At present the authors can just state that there is a co-immunoprecipitating protein being phosphorylated at threonine residues. Since several kinases are always co- immunoprecipitating they can¥t assume that the phosphorylation is mediated by CK1e. IP in combination with kinase assays in the presence and absence of CK1 specific inhibitors respectively will provide first evidence that co-immunoprecipitating proteins are phosphorylated by CK1e. Furthermore, it is not obvious from scansite analysis that ANT2 is a target for CK1e, whereas scansite analysis revealed that T160 and T230 of Prohibitin could be potential phosphorylated by CK1e. Furthermore, it seems that additional serine residues of Prohibitin could be targeted by CK1e. Additional experiments are necessary to show that Prohibitin (and/or ATN2) are phosphorylated by CK1e in vitro and in vivo.

Analysis of the CK1e kinase activity in the different cell lines as well as identifying Prohibitin and/or ATN2 as CK1e substrates would increase the stringency of the paper and should therefore be performed.

Referee #3:

Authors made several additional experiments that now better support the interpretation of the results, notably regarding the partners of CKI epsilon.
Therefore, I recomend the manuscript for publication.

2nd Revision - Authors' Response 07 May 2012

We are very pleased that all of the reviewers were satisfied with our revised manuscript except the first referee, who has requested more data and rewriting. We have performed the CKIa IHC and have carefully taken advice of the referees and rewritten the manuscript. We do not have enough materials and time to finish an extensive phosphorylation study of CKIe substrates within the constraints of Journal’s time limit. We have spent a lot of efforts to improve the quality of figures and added a Table of Content for the SI according to your suggestions. However, as the limit for SI is only 10 MB, we can only submit a low-resolution version of SI.

We are addressing the first reviewer’s comments as follows:

***** Reviewer's comments *****

Referee #1:

In the revised version Rodriguez and co-workers have addressed all issues which have been raised by the reviewers, although some questions are still not answered satisfactorily. Nevertheless the stringency of the manuscript has increased. At present the main weakness of the manuscript is the description and interpretation of the CK1 expression data and inhibitor experiments. Rewriting and more carefully interpretation of these results seems to be mandatory. Furthermore, the

phosphorylation data are preliminary.

Analysis of the expression levels of CK1 isoforms: page 5: "While ovarian tumours stained positive for CK1e, ...weak to negative staining for CK1d, confirming that the positive staining comes from CKIe alone." I agree that the expression of CK1 isoforms differ in the tumours, but not that the staining comes from CK1e alone. Whereas in most cases low and high scores for CK1e expression was counted, the expression of CK1d was not detected or expressed very weakly. Furthermore, I would assume that CK1a is expressed differently in tumours presumably showing high expression levels in many tumours (therefore, showing IHC analysis for CK1a would increase the stringency of the paper). The author could state that CK1 isoforms are differently expressed in ovary tumours.

"CK1d, and the more distant CK1a expression in ovarian cell lines showed almost absence of CK1d expression and no significant overexpression of CK1a in the ovarian cancer cell lines".... Western blot results suggest that only the CK1e isoform is overexpressed...". The data presented in figure S2 clearly show, elevated expression of CK1d in one ovarian tumour cell line (Ovca 420). Furthermore, the expression levels of CK1a differ in the analyzed tumour cell lines. Whereas CK1a expression is down-regulated in 5 cases it is up-regulated in 3 cases (that means CK1e is not the only isoform up-regulated in ovarian tumour cell lines) (therefore, additional IHC analysis of CK1a expression in tumour samples might be of interest, see above).

We thank Referee #1 for the persistence in evaluating the expression patterns of different CKI isoforms. First, we have performed IHC for CKIa and added the results to Figure S1D. The results showed that this isoform is expressed in almost all types of cells in ovarian tissues. The expression in the stromal fibroblasts was significant, albeit they were weaker than the epithelial cells. The normal ovarian surface epithelial cells stained at least equally as, if not stronger than, many of the epithelial tumour cells. This is consistent with the Western blot analysis of this isoform in ovarian epithelial cell lines in Figure S2. Even though there were two cancer cell lines that showed higher expression of CKIa than the normal HOSE cells, the other six cancer cell lines either showed similar or weaker CKIa staining than the HOSE cell lines. Hence, we think that the CKIa is not significantly overexpressed in ovarian tumours relative to the normal HOSE cells. Second, we have used two CKIe antibodies and also peptide pre-absorption in the IHC (Figure S1B), stained cancer cell lines prepared as paraffin-embedded tissues (Figure S1C) to validate the specificity of the CKIe IHC staining. All the staining of the CKIa, CKId, and CKIe isoforms in IHC (Figure 1A, Figure S1) and Western (Figure 1C and Figure S2) showed completely different patterns. Therefore, the isoform antibodies did detect only their respective isoforms and did not cross-react with other isoforms. Given all these data, we are very sure that the results of CKIe staining were from the CKIe isoform only and therefore do not understand why the referee #1 does not agree that the CKIe staining was not from CKIe alone (first underlined sentence in the review above).

In response to whether CKIe is the only isoform significantly overexpressed in ovarian tumours, we have to clarify that all expressions in the tumours are compared relative to normal ovarian surface epithelial cells. We should agree that the CKId isoform is definitely not significantly overexpressed in ovarian cancer cells (only 1 out of 8 cancer cell lines in Figure S2 showed moderate expression, and which was seen only after prolonged exposure). For CKIa, only 2 out of 8 cancer cell lines in Figure S2 showed higher expression than the normal HOSE lines, and given the fact that CKIa stained very strongly in normal ovarian surface epithelium in IHC (Figure S1D), we do not think that CKIa is significantly overexpressed in ovarian tumours relative to normal ovarian surface epithelial cells. In contrast, CKIe IHC staining in normal ovarian surface epithelium is very weak (compare the staining of normal ovary in Figure 1A with that of Figure S1D) compared with the malignant tumours. In Western blot, 15 out of 17 cancer cell lines in Figure 1C, and 7 out of 8 cancer cell lines in Figure S2 showed higher CKIe expression than the normal HOSE lines. Based on all the IHC and Western blot data, we want to emphasize that there are other CKI isoforms such as CKIa that are expressed in ovarian tumours besides CKIe, but the CKIe isoform is likely the CKI isoform that is significantly overexpressed in ovarian tumours compared with normal human ovarian surface epithelial cells. We have made the necessary amendments in the Results Section.

The authors should also indicate in all figure legends which primary antibody had been used.

We have made the changes as requested by the referee.

"Hence, it is apparent that over-expression of wild - type CK1e transcript occurs in ovarian cancer". Since the authors did not sequenced all exons of CK1e the interpretation of their sequence results have to be more carefully; they can´t exclude the presence of mutations in those exons which have not been analyzed.

We have made the changes as requested by the referee.

The CK1 inhibitor chapter (page 6/7) needs some rewriting and more carefully interpretation of the results. "Three pharmacological inhibitors of CK1e..." Note, all three inhibitors inhibit CK1 isoforms, some of them with higher affinity towards CK1d/e or CK1e. "Both treatments with PF670462 and PF-4800567 caused a slight increase in G1 phase...". The data presented in figure S5 are not convincing. Upon PF670462 treatment no G1 arrest is visible and also the effects of PF-4800567 are negligible. Furthermore, the cell cycle analyses for those two inhibitors are not in line with the results of the MTT assays. Furthermore, not all cell lines used for MTT analyses are mentioned in the legend of figure 3a (e.g. violet line in the PF670462 graph)

We agree that the pharmacological inhibitors have different affinity towards CKId/e. In Figure S5, it is true that the effect of IC261 was much more obvious than the other two inhibitors. The FACS results were treatment of inhibitors overnight, whereas for MTT, the treatment lasted for more than 48 hours. Different cell lines also showed different responses.

In the MTT figure (Figure 3A), the same six cancer cell lines were used for different CKI inhibitors. There was a mistake in presenting the TOV112D line for the PF670462 graph. We have corrected the figure.

Finally the phosphorylation data presented are not convincing. First at all, the question 2b having been raised by referee 1 has not been answered. Instead a complete other aspect was addressed. The authors now wanted to know if the CK1e interacting mitrochondrial proteins (ANT2 and/or Prohibitin) are phosphorylated by CK1e. To solve this question additional experiments are necessary. At present the authors can just state that there is a co-immunoprecipitating protein being phosphorylated at threonine residues. Since several kinases are always co- immunoprecipitating they can´t assume that the phosphorylation is mediated by CK1e. IP in combination with kinase assays in the presence and absence of CK1 specific inhibitors respectively will provide first evidence that co-immunoprecipitating proteins are phosphorylated by CK1e. Furthermore, it is not obvious from scansite analysis that ANT2 is a target for CK1e, whereas scansite analysis revealed that T160 and T230 of

Prohibitin could be potential phosphorylated by CK1e. Furthermore, it seems that additional serine residues of Prohibitin could be targeted by CK1e. Additional experiments are necessary to show that Prohibitin (and/or ATN2) are phosphorylated by CK1e in vitro and in vivo.

Analysis of the CK1e kinase activity in the different cell lines as well as identifying Prohibitin and/or ATN2 as CK1e substrates would increase the stringency of the paper and should therefore be performed.

We thank Referee #1's criticism and suggestions. We repeat here that we did not have the permit to do the kinase assay for Question 2b. We therefore had explored to determine the phosphorylation status of its interacting proteins, which can serve as a readout of kinase activity. As shown in Figure S8B, there was a strong reactivity of protein(s) to the phospho-threonine antibody at the position where ANT2 and prohibitin were located in the CKIe immunoprecipitation lane. We therefore suspect that it might be derived from the phosphorylation of either one or both of these proteins. We are planning to perform a large-scale phosphoprotein identification mediated by CKIe using the mass spectrometry-based platform. However, the experiment is too extensive to be finished in a two-

week time period. There are definitely a lot of experiments that can be done to further investigate the details of CKIe-mediated downstream pathways and the consequences on tumour development. However, we feel that there is a limit in terms of time and efforts to complete the manuscript in this stage, as suggested by the Editor of the Journal.

Referee #3:

Authors made several additional experiments that now better support the interpretation of the results, notably regarding the partners of CKI epsilon.

Therefore, I recommend the manuscript for publication.

We thank deeply Referee #3 for the total acceptance of our extensive efforts in revising the manuscript.

We greatly appreciate the thoughtful and constructive comments of all referees. We have thoroughly and clearly responded to all of their constructive comments by performing additional experiments and rewritten the manuscript. We strongly feel that this second revised manuscript is substantially strengthened by our responses to reviewers' comments. We hope that we will now receive a favorable decision regarding publication of this version of manuscript soon.

© EMBO
