# Supplementary material for: Casein kinase I epsilon interacts with mitochondrial proteins for the growth and survival of human ovarian cancer cells
Source: EMBO Mol Med. 2012 Jun 18;4(9):952–63. doi: 10.1002/emmm.201101094 (PMC3491827; doi:10.1002/emmm.201101094)
Supplement: Supplementary file 2 [file emmm0004-0952-SD2.pdf]

Supplementary Information

Table of Content

Page number

SI Materials and Methods 1-9

SI Supplementary Figures

Figure S1. Immunohistochemistry staining for the CKI isoforms in ovarian tissues. **10**

Figure S2. Expression of CKI isoforms in ovarian cell lines. **11**

Figure S3. Sequencing of *CKIε* gene in ovarian tumors and cancer cell lines. **12**

Figure S4. Overexpression of CKIε in normal HOSE cells increased spheroid size. **13**

Figure S5. Cell cycle distribution of ovarian cancer cells treated with CKI inhibitors. **14**

Figure S6. Characterization of xenograft tumors. **15**

Figure S7. Peptide mass analysis results of the Prohibitin, ANT2, Annexin A2, and CKIε proteins. **16**

Figure S8. Reciprocal immunoprecipitation assays and phosphorylation status of immunoprecipitated proteins. **17**

Figure S9. Subcellular distribution of CKIε and the three mitochondrial proteins in the control and CKIε knockdown (KD) cell lines. **18**

Figure S10. ANT2 mediates the effects of CKIε on ovarian cells. **19**

SI Supplementary Tables

Table S1. Means and Medians for Survival Time (Positive CKIε versus Negative CKIε) **20**

Table S2. Log rank test for comparing survival (Positive CKIε versus Negative CKIε) **20**

SI MATERIALS AND METHODS

Immunohistochemistry

Paraffin embedded ovarian tissue blocks were sectioned at a thickness of 7 μm paraffin-embedded ovarian tissue sections were mounted on Superfrost Plus microscopic slides (Fisher Scientific, Pittsburgh, PA), and dried at 50°C for at least 24 hours. Deparaffinization was performed using xylene and rehydration with a graded ethanol series. Antigen retrieval was performed in a pressure-cooker in antigen-unmasking solution (Vector Laboratories, Burlingame, CA) for 10 min. Endogenous peroxidases were blocked using 0.3% H_20_2 in methanol for 20 min. The sections were then blocked with normal horse serum for 20 min and were subsequently incubated overnight with the primary antibody. After incubation, the reaction was visualized using Vectastain Elite ABC Kit with diaminobenzidine chromogen as a substrate (Vector Laboratories, Burlingame, CA). Sections were counterstained lightly with hematoxylin and mounted in Permount (Fisher Scientific, Pittsburgh, PA). The staining was quantified with a semiquantitative scoring system. The weighted score was obtained by multiplying the staining intensity score (3+, strong positive stain in cells; 2+, moderate stain in cells; 1+, weak stain in cells; 0, no evidence of stain) and score for the percentage of positive cells (3+, most of cells stained; 2+, half of cells stained; 1+, few cells stained; 0, no cells stained). Two trained observers scored the slides independently, and the scores for all cases were compared for discrepancies.

Lentiviral Infection, siRNA and DNA transfection

Mission™ lentiviral CKIε-targeting and non-target control shRNA transduction particles and Mission siRNAs were purchased from Sigma-Aldrich (St. Louis, MO). 1 x 10^5 MCAS and SKOV3-IP^{Luc} ovarian cancer cells growing in complete medium were infected with 5 x 10^5 transduction particles in the presence of 8 μg/ml hexadimethrine bromide (Sigma-Aldrich, St. Louis, MO) and incubated overnight at 37°C and 5% CO_2. On the third day, medium was replaced with complete medium containing 2 μg/ml

puromycin and changed every 3 days for 2 weeks. Knockdown of CKIε expression in the resultant cell lines was confirmed by Western blot analysis. For the introduction of CKIε into HOSE cells, full-length CKIε cDNA expression construct and empty vector purchased from OriGene Technologies (Rockville, MD) were transfected separately into the HOSE cells using Lipofectamine™ 2000 transfection reagent (Invitrogen Corp. Carlsbad, CA). The transfected cells were selected using complete medium containing 500 μg/ml G418 (Invitrogen Corp. Carlsbad, CA) and stable clones with CKIε expression were confirmed by Western blot analysis. siRNAs were transfected into MCAS and SKOV3 cancer cells using Lipofectamine™ 2000 transfection reagent and the transfected cells were analyzed after two days.

Characterization of cell lines with changes of CKIε expression

(A) Cell growth and migration assays

Cell proliferation was determined by seeding 3 x 10^4 cells to 35-mm culture dishes and allowed to grow to different time points. At each time point, the number of cells in three culture dishes were counted and averaged for each cell line. Cell migration was evaluated in Transwell inserts with 8 μm pore-sized PET membranes (BD Biosciences, San Jose, CA) coated with 10 μg/ml collagen (Sigma-Aldrich, St. Louis, MO) overnight. The following day, 1×10^5/ mL cells in serum-free medium containing 0.05%BSA were added to the inserts. The inserts were then placed in the wells of a companion plate and incubated with complete medium containing 10% fetal bovine serum in the lower chamber. After 24 hours of incubation, the cells in the inserts were scraped with cotton swaps. The membranes were excised from the inserts and counterstained with Diff-Quik® Stain Set (Dade Behring Inc., Newark, DE). The cells that migrated through the membrane were counted in four random fields. Each experiment was repeated twice and average values ± standard errors are presented.

(B) Luciferase reporter assay to measure β−catenin-mediated transcriptional activation

MCAS cell lines with changes in CKIε expression, as well as the HEK/293 control cell line, were co-transfected with the *pTOPFLASH* vector (Addgene, Cambridge, MA) that contains multimers of β-catenin/TCF binding sequences upstream of the firefly luciferase reporter gene, and a *Renilla* luciferase-encoding plasmid *pRL-CMV* (Promega, Madison, WI) as internal control. Another transfection with a *pFOPFLASH* (Addgene, Cambridge, MA) plasmid that harbors mutant β -catenin/TCF-binding sites together with *pRL-CMV* plasmid was performed to serve as negative control. As a positive control for the Wnt-signaling pathway mediated by β-catenin activity, HEK/293 control cells were treated with 5 mM LiCl (Sigma-Aldrich Corporation, St. Louis, MO, USA), a Wnt signaling activator by GSK-3β inhibition, for 24 hours and harvested. Cell suspensions after lysis were centrifuged at 12,000 x g. Firefly and *Renilla* luciferase activities of cell lysates were measured using the Dual Luciferase Assay System (Promega, Madison, WI) in a Turner TD-20/20 luminometer (Turner Designs, Sunnyvale, CA, USA). The relative firefly luciferase activity was normalized to Renilla luciferase activity for each cell type. The results were presented from four independent experiments. The results are reported as means ± standard errors.

(C) MTT cytotoxicity assay and cell cycle determination

Cell suspensions (7 x 10^4 cells/mL) were seeded in 100 μl of medium per well in 96-well plates. After overnight incubation, 100 μl of medium with serial dilutions of CKIε inhibitor IC261 (Santa Cruz Biotech, Santa Cruz, CA), PF-670462 (Tocris Bioscience, Ellisville, MO), and PF-4800567 (R&D Systems, Minneapolis, MN), as well as chemotherapeutic agents carboplatin, and paclitaxel (Sigma-Aldrich, St. Louis, MO) were added for 48 hr. 20 μL of MTT solution (5 mg/mL in PBS, Sigma-Aldrich, St. Louis, MO) was added to each well and incubated for 4 hours. Medium was aspirated and 100μL of combined solubilization solutions, 10% SDS in 0.01N HCl, and 0.1N HCl in 2-propanol (1:1), was added to each well. Absorbance at 562 nm was determined on an ELx800 absorbance microplate reader (Bio-Tek, Winooski, VT). Each drug assay was performed in triplicates and repeated twice.

For cell cycle determination, the cells were treated overnight separately with 10 μM of PF670462, 50 μM of PF4800567, and 5 μM of IC261. Control cells were treated with equal volume of vehicle DMSO. On the second day, cells were trypsinized, washed in PBS, and resuspended in ice-cold 70% ethanol for 30 minutes and then stained with propidium iodide (50 μg/ml) in the presence of RNase A (200 μg/ml) (Sigma-Aldrich, St. Louis, MO). Samples were analyzed using Accuri C6 Flow Cytometer (BD Accuri Cytometers, Ann Arbor, MI).

Mouse xenograft experiment

5×10^6 SKOV3-IP^{Luc} cells harboring control shRNA or shRNA construct 3 were injected into the peritoneal cavity of 12 5-6 week old athymic female nude mice (Taconic, Hudson, NY). The mice were kept at the animal facility under standard conditions at Brigham and Women's Hospital for 28 days, at which time whole-body bioluminescent images were taken. For the imaging, mice were administered intraperitoneally with D-luciferin (150 mg/kg body weight), waited for 10 minutes, and placed in a clear Plexiglas anesthesia box with 2.5-3.5% isofluorane. After the mice were fully anesthetized, they were transferred to the nose cones attached to the manifold in the imaging chamber of a Xenogen IVIS-Imaging System 100 Series (Caliper LifeSciences, Hopkinton, MA). Whole-body bioluminescent images were acquired and analyzed using the Living image 2.60 software (Caliper LifeSciences, Hopkinton, MA). The mice were sacrificed and the tumors were harvested and weighed. Parts of the tumors were formalin-fixed and paraffin-embedded for immunohistochemical staining. Parts of the tumors were fresh frozen and lysates were prepared for Western blot analysis.

Co-Immunoprecipitation and protein identification

Wild-type MCAS cells were lysed in CoIP buffer (50 mM Tris, 150 mM NaCl, 50 mM NaF, 0.5% Nonidet P-40, 1 mM PMSF, 1 mM DTT, 0.5 mM Na_3VO_4, 10 mM β-glycerophosphate, 2 mM sodium molybdate, pH 7.5, and 10% glycerol). 500 μg of cleared lysates after centrifugation were incubated at 4 °C overnight with specific antibody or a control antibody, followed by a 2-hour incubation with a 50:50 mixture of protein A/G immobilized on agarose beads (Pierce Biotechnology, Rockford, IL). The beads were pelleted, washed four times with CoIP buffer, and the precipitated proteins were separated by sodium dodecyl sulphate polyacrylamide gel electrophoresis (SDS-PAGE). The resolved proteins were transferred from the gel to PVDF membrane (Pierce Biotechnology, Rockford, IL) and probed with specific antibody using Western blot analysis. To identify unknown proteins, the gel was stained using the sensitive Colloidal Coomassie universal protein dye (Invitrogen Corp. Carlsbad, CA). The band for the protein of interest was excised from the gel and sent to the Taplin Biological Mass Spectrometry Facility at Harvard Medical School for protein identification by mass spectrometry.

Immunofluorescence microscopy

Wild-type MCAS cells were fixed in 4% paraformaldehyde (Sigma-Aldrich, St. Louis, MO) and permeabilized with PBS containing 0.5% Triton X-100 (Sigma-Aldrich, St. Louis, MO). After blocking with 10% fetal bovine serum (Invitrogen, Carlsbad, CA), primary antibodies were added and incubated at 24 ºC for 2 hours. After washing, anti-mouse antibody coupled with Alexa Fluor 647 and Alexa Fluor 546-conjugated secondary antibodies (Invitrogen, Carlsbad, CA) were used to stain CKIε, and mitochondrial proteins, respectively. The stained cells were counterstained with Sytox Green (Invitrogen, Carlsbad, CA). Microscopic images were captured by Leica SP5 confocal microscope (Leica Microsystems, Bannockburn, IL) and analyzed by the Leica LAS AF software (Leica Microsystems, Bannockburn, IL).

Subcellular protein fractionation

Nuclear and cytosolic fractions were isolated according to Ng *et al* (42). $1x10^8$ of MCAS shRNA control and CKIε knockdown cells were resuspended in Buffer A (10 mM HEPES, pH 7.9, 1.5 mM $MgCl_2$, 10 mM KCl, and 0.5 mM DTT) with 0.1% Nonidet P-40 on ice for 10 min. After centrifugation at 600 x g, the supernatant was removed and centrifuged further at 100,000 x g to obtain clear cytosolic fraction. The pellet was resuspended in 150 μl of Buffer C (20 mM HEPES, pH 7.9, 0.42 M NaCl, 1.5 mM $MgCl_2$, 0.2 mM EDTA, 0.5 mM PMSF, 0.5 mM DTT, and 25% (v/v) glycerol) and incubated on ice for 30 min, sonicated and centrifuged at 12,100 x g for 10 min. Supernatant was transferred to 750 μl of Modified Buffer D (20 mM HEPES, pH 7.9, 0.05M KCl, 0.2 mM EDTA, 0.5 mM PMSF, 0.5 mM DTT, and 20% (v/v) glycerol), mixed to give the nuclear fraction. Mitochondria were isolated according to Frezza *et al* (43). In brief, $1x10^8$ of cells were homogenized respectively in 1.5 ml of ice-cold **IB_c** buffer (0.2 M sucrose, 10 mM Tris-MOPS, pH 7.4, 1 mM EGTA). After centrifugation at 600 x g for 10 min, the supernatants were centrifuged at 7,000 x g for 10 min at 4 ^oC. The pellets were washed once with 200 ul of ice-cold IBc and re-centrifugation. The mitochondrial pellets were resuspended in 100 ul of 1xRIPA lysis buffer (50 mM Tris HCl pH 8, 150 mM NaCl, 1% NP-40, 0.5% sodium deoxycholate and 0.1% SDS).

Western blot

Total cell lysates were prepared from growing cells using RIPA buffer supplemented with PhosStop phosphatase inhibitor cocktail and complete protease inhibitor cocktail (Roche Applied Science, Indianapolis, IN) and protein concentration was measured with a MicroBCA protein assay kit (ThermoScientific, Rockford, IL). 10 μg of total cell lysates of the cell lines were resolved by sodium dodecyl sulfate-polyacrylamide gel electrophoresis (SDS-PAGE) and transferred to a polyvinylidene fluoride (PVDF) membrane using a SEMI-DRY Transfer cell (Bio-Rad Laboratories, Hercules, CA). After blocking with 5% nonfat dry milk in 1X TBST buffer (10mM Tris-HCl pH 7.5, 150 mM NaCl, 0.05% Tween-20) at room temperature for 1 hr, the membrane was incubated with a primary antibody at

4ºC overnight then washed at room temperature. The bound antibody was detected by a horseradish peroxidase-conjugated secondary antibody (Pierce Biotechnology, Rockford, IL) and a Supersignal west pico kit (Pierce Biotechnology, Rockford, IL). The CKIε signals were quantified using a GS-700 Imaging Densitometer (Bio-Rad Laboratories, Hercules, CA) and normalized to β-actin signals.

ATP assay

Intracellular ATP was measured using an ATP Bioluminescent Assay kit (Sigma-Aldrich, St. Louis, MO), which is based on the dependence of the luminescence output of the luciferase-catalyzed oxidation of exogenous luciferin substrate on intracellular ATP concentration of the cell lysates. Cell lysates were prepared using Passive Lysis Buffer (Promega, Madison, WI) and the luminescence was measured in a Turner TD-20/20 luminometer (Turner Designs, Sunnyvale, CA, USA).

Statistical Analysis

All calculations were performed with MINITAB statistical software (Minitab, State College, PA) unless otherwise indicated. ANOVA was used to compare the mean IHC scores between benign and malignant paraffin sections and between different tumor histologies. Significance of the test was considered at the 5% level (i.e. P-value ≤ 0.05). For the survival analysis, the length of overall survival was defined from the date of operation to the date of the patient's death (uncensored) or the date of last visit (censored). We defined the low and high CKIε expression groups using the cutoff score given by the median score of all samples (Score 3.0). The choice of cutoff score was based on the ability to group cases with significant differences in overall survival. Overall survivals of patients with positive and negative CKIε expression were estimated using the Kaplan-Meier method, and compared with a log rank test. The impact of CKIε expression on patient survival was further studied with the inclusion of potential clinical risk factors, using the Cox proportional hazards regression model. The variables applied for adjustment in the multivariate analysis included tumor grade, stage of disease, CA125 values, optimal or suboptimal

debulking, and patient's age. Patient's age was treated as a continuous variable, while tumor grade and stage of disease were analyzed as ordinal variables. Grade 1 was considered as the reference group to identify the risk of death of combination of grades 2 and 3 cases. Stage 1 and 2 cases were grouped as the reference group to evaluate the risk of death of the combination of stage 3 and 4 cases. With the multivariate Cox proportional hazards analysis, the variables were entered into the model using a forward stepwise Wald-test process. The impact of CKIε expression on patient survival was determined by examining the relative hazard ratios with respect to the positive/negative CKIε expression groups in the final model. The significance of estimated hazard ratios was tested using the Wald test. A significant result implies a difference in overall survival between the positive and negative CKIε expression groups after the adjustment for other risk factors. The Cox regression analysis was performed using the SPSS 17.0 statistical software. (SPSS, Inc, Chicago, IL).

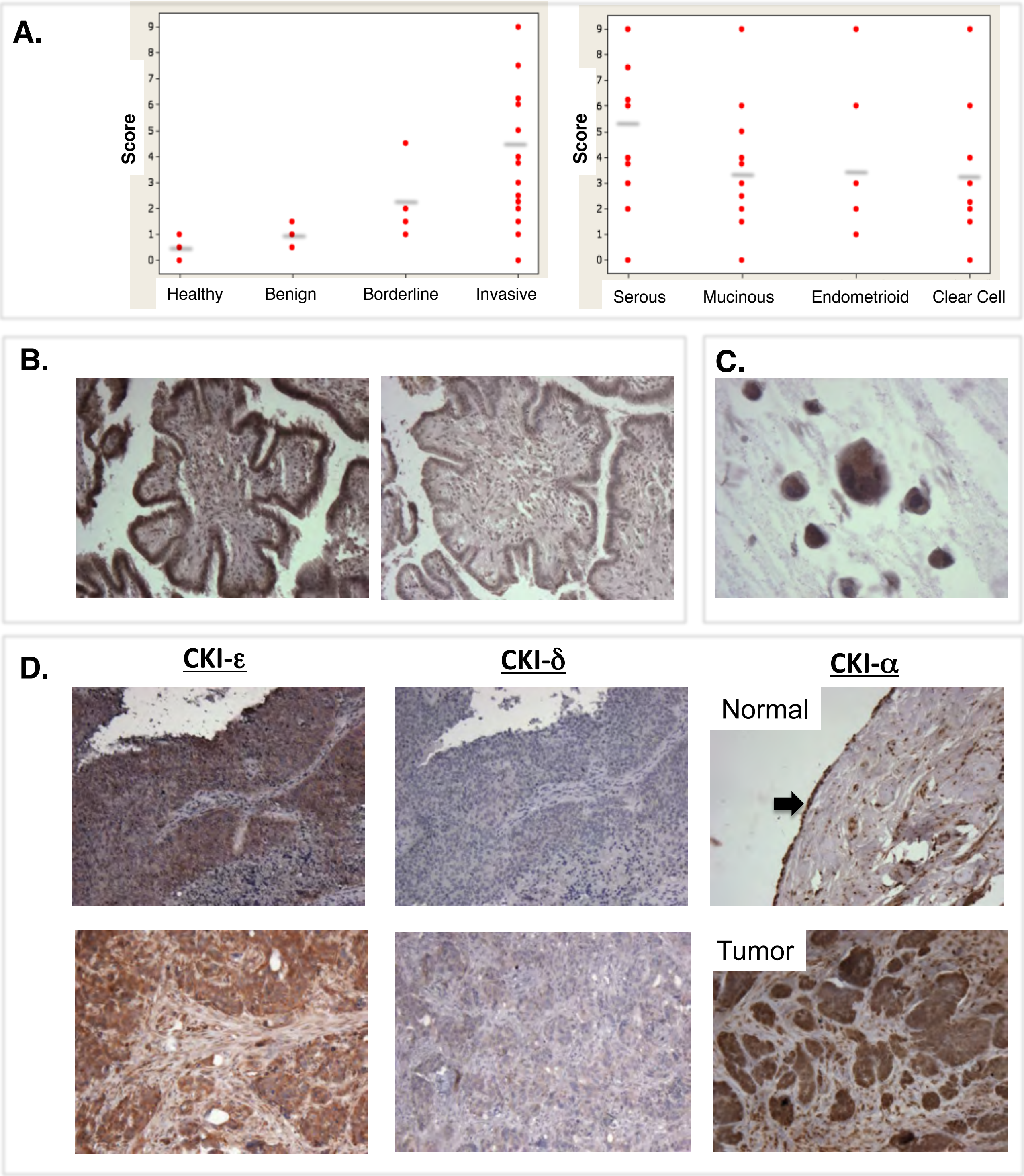

Figure S1. Immunohistochemistry staining for the CKI isoforms in ovarian tissues.
A. Scatter plots to show the staining of CKIε among different diagnostic (left) and histologic (right) groups, with bars indicating the mean scores. **B.** Representative immunohistochemical images of using a second CKIε-specific antibody from Abgent (left) and neutralization of CKIε-specific antibody with CKIε blocking peptides. **C.** Immunohistochemical staining of CKIε in SKOV3 ovarian cancer cells after fixation and paraffin-embedding procedure as the tissue specimens. **D.** Comparison of staining for CKIε and CKIδ isoforms in two cases of serous ovarian tumors demonstrated elevated expression of CKIε in ovarian tumors. Equal CKIα staining in normal ovarian surface epithelium (arrowhead) and in tumor tissues.

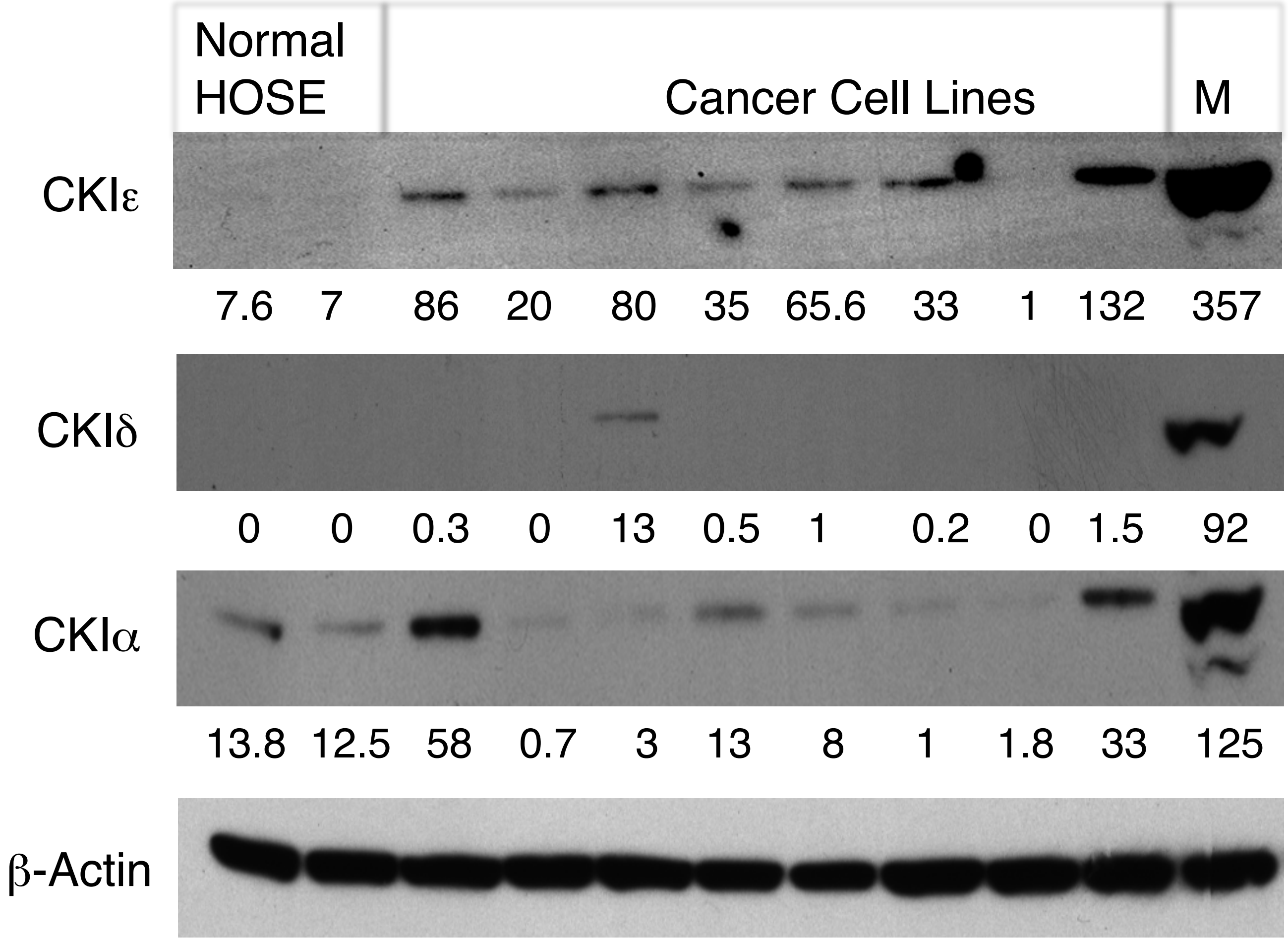

Figure S2. Expression of CKI isoforms in ovarian cell lines.
Western blot analysis of normal HOSE cell lines and ovarian cancer cell lines for CKI isoforms. The CKIε antibody was from BD Biosciences. From left to right: HOSE 1-15, HOSE 642, OVCA810, RMG1, OVCA420, OVCA432, SKOV3, MCAS, RMUGL, TOV112D. Mouse brain lysates (M) was used as positive control for the CKI isoforms. The signal intensities of the isoforms were normalized to β-actin signals for each cell line.

A.

```
> ref|NW_001838745.1| Homo sapiens chromosome 22 genomic contig, alternate assembly
HuRef SCAF_1103279188372, whole genome shotgun sequence Length=21026802  Features in
this part of subject sequence:
casein kinase I isoform epsilon
casein kinase I isoform epsilon
Score =  865 bits (468),  Expect = 0.0
Identities = 471/472 (99%), Gaps = 1/472 (0%)
Strand=Plus/Minus

Query  12       CCCGGGCAGGTGCC-ACATCGCCTCTGGTGAGGAAGTCGCCATCAAGCTGGAGTGTGTGA  70
                |||||||||||||| |||||||||||||||||||||||||||||||||||||||||||||
Sbjct16792784   CCCGGGCAGGTGCCAACATCGCCTCTGGTGAGGAAGTCGCCATCAAGCTGGAGTGTGTGA  16792725
Query  71       AGACAAAGCACCCCCAGCTGCACATCGAGAGCAAGTTCTACAAGATGATGCAgggtggcg  130
                ||||||||||||||||||||||||||||||||||||||||||||||||||||||||||||
Sbjct16792724   AGACAAAGCACCCCCAGCTGCACATCGAGAGCAAGTTCTACAAGATGATGCAGGGTGGCG  16792665
Query  131      gtgagcgcagggtgggcggcggcgggcgccgggtgggtggcggtgggcAGCCATGAGCAC  190
                ||||||||||||||||||||||||||||||||||||||||||||||||||||||||||||
Sbjct16792664   GTGAGCGCAGGGTGGGCGGCGGCGGGCGCCGGGTGGGTGGCGGTGGGCAGCCATGAGCAC  16792605
Query  191      AGGGTGGGCCGGGCGTGGCTGGCTGCCTCTGACCCCTGACAGAGGCCCTCTGCTTGACCC  250
                ||||||||||||||||||||||||||||||||||||||||||||||||||||||||||||
Sbjct16792604   AGGGTGGGCCGGGCGTGGCTGGCTGCCTCTGACCCCTGACAGAGGCCCTCTGCTTGACCC  16792545
Query  251      CCAGTGGGGATCCCGTCCATCAAGTGGTGCGGAGCTGAGGGCGACTACAACGTGATGGTC  310
                ||||||||||||||||||||||||||||||||||||||||||||||||||||||||||||
Sbjct16792544   CCAGTGGGGATCCCGTCCATCAAGTGGTGCGGAGCTGAGGGCGACTACAACGTGATGGTC  16792485
Query  311      ATGGAGCTGCTGGGGCCTAGCCTCGAGGACCTGTTCAACTTCTGTTCCCGCAAATTCAGC  370
                ||||||||||||||||||||||||||||||||||||||||||||||||||||||||||||
Sbjct16792484   ATGGAGCTGCTGGGGCCTAGCCTCGAGGACCTGTTCAACTTCTGTTCCCGCAAATTCAGC  16792425
Query  371      CTCAAGACGGTGCTGCTCTTGGCCGACCAGATGGTGAGTCCCCGTCCCCTCCATCCCCAG  430
                ||||||||||||||||||||||||||||||||||||||||||||||||||||||||||||
Sbjct16792424   CTCAAGACGGTGCTGCTCTTGGCCGACCAGATGGTGAGTCCCCGTCCCCTCCATCCCCAG  16792365
Query  431      CCAGATGCCTGTGGGCCCAGGATACCAGCCCTGCCTCCTGCCCCCAGAGGAT  482
                ||||||||||||||||||||||||||||||||||||||||||||||||||||
Sbjct16792364   CCAGATGCCTGTGGGCCCAGGATACCAGCCCTGCCTCCTGCCCCCAGAGGAT  16792313
```

B.

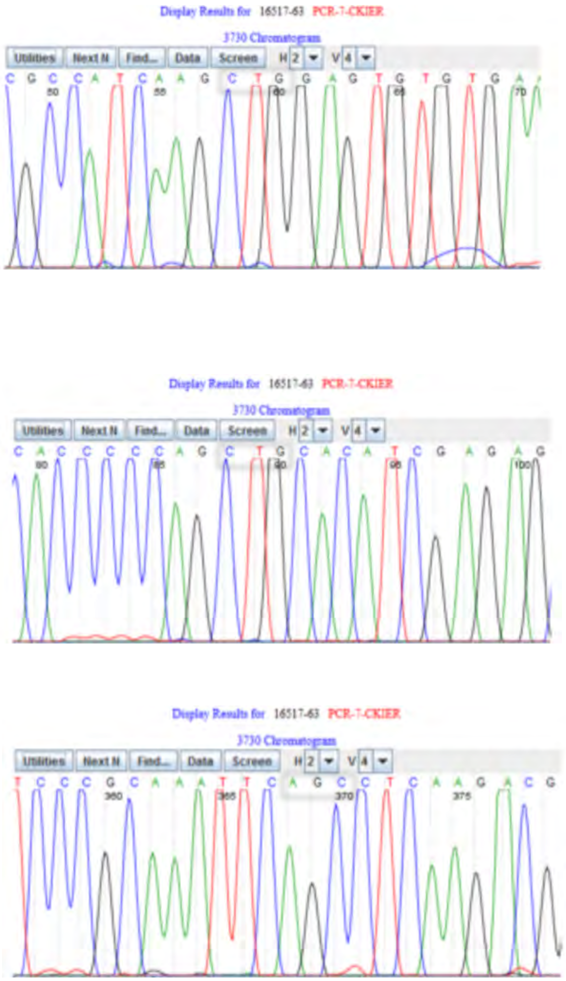

Figure S3. Sequencing of *CKIε* gene in ovarian tumors and cancer cell lines.
A. Representative alignment of a PCR fragment amplified from a serous cancer genome for CKIε to the GenBank human RefSeq Genomic dataset using the BLAST program. Yellow and red boxes represent the multiple sites where somatic mutations were identified by Fuja *et al*. **B.** Representative images of sequencing chromatograms for the red box regions in **A**, indicating the wild-type sequences in the sequenced ovarian samples.

A.

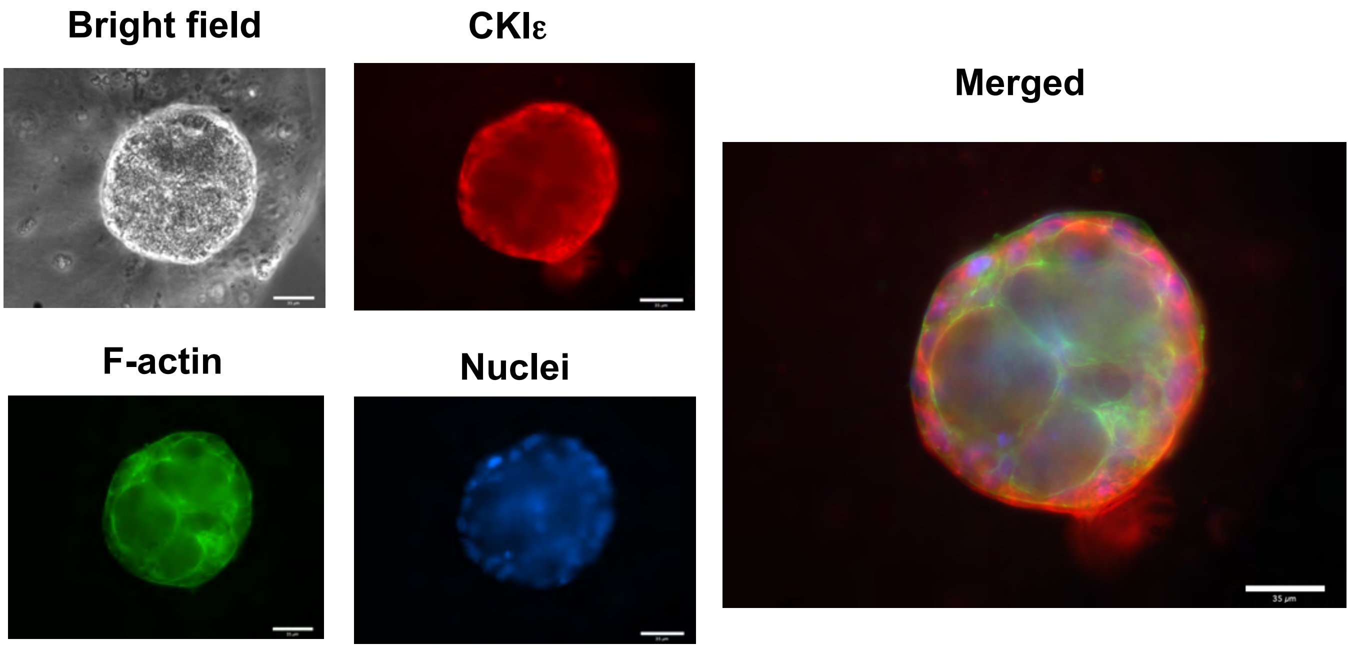

B.

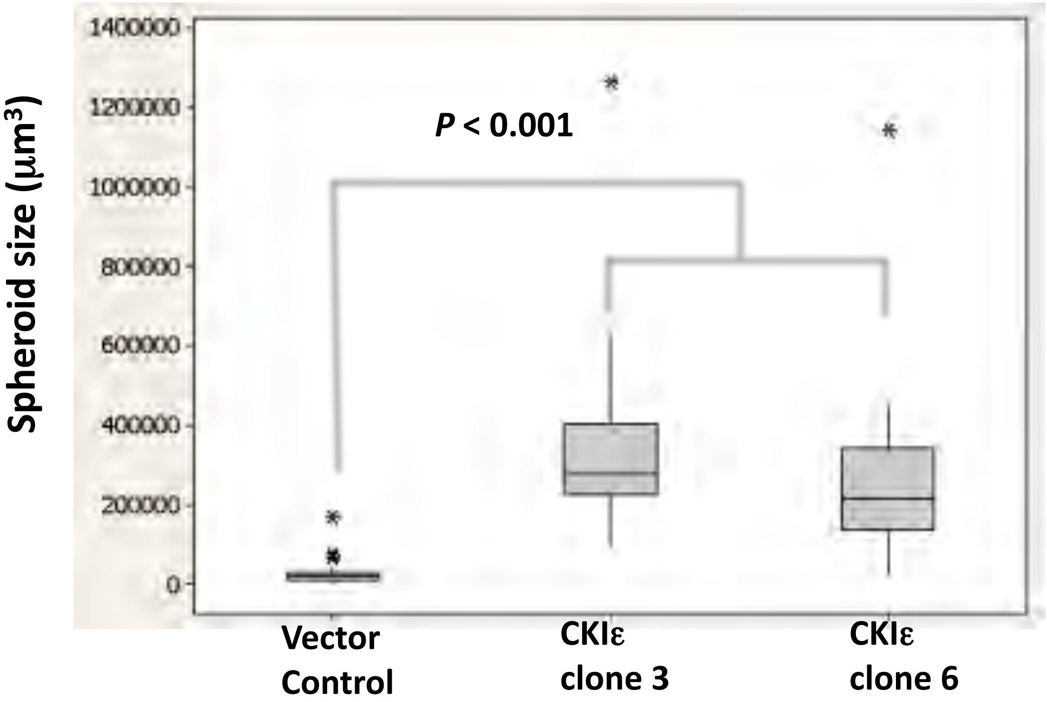

Figure S4. Overexpression of CKIε in normal HOSE cells increased spheroid size.

A. Immunofluorescence to show the expression of CKIε in the cytoplasm of cells lining the lumen. **B.** Comparison of spheroid sizes between control and CKIε-overexpressing HOSE cells.

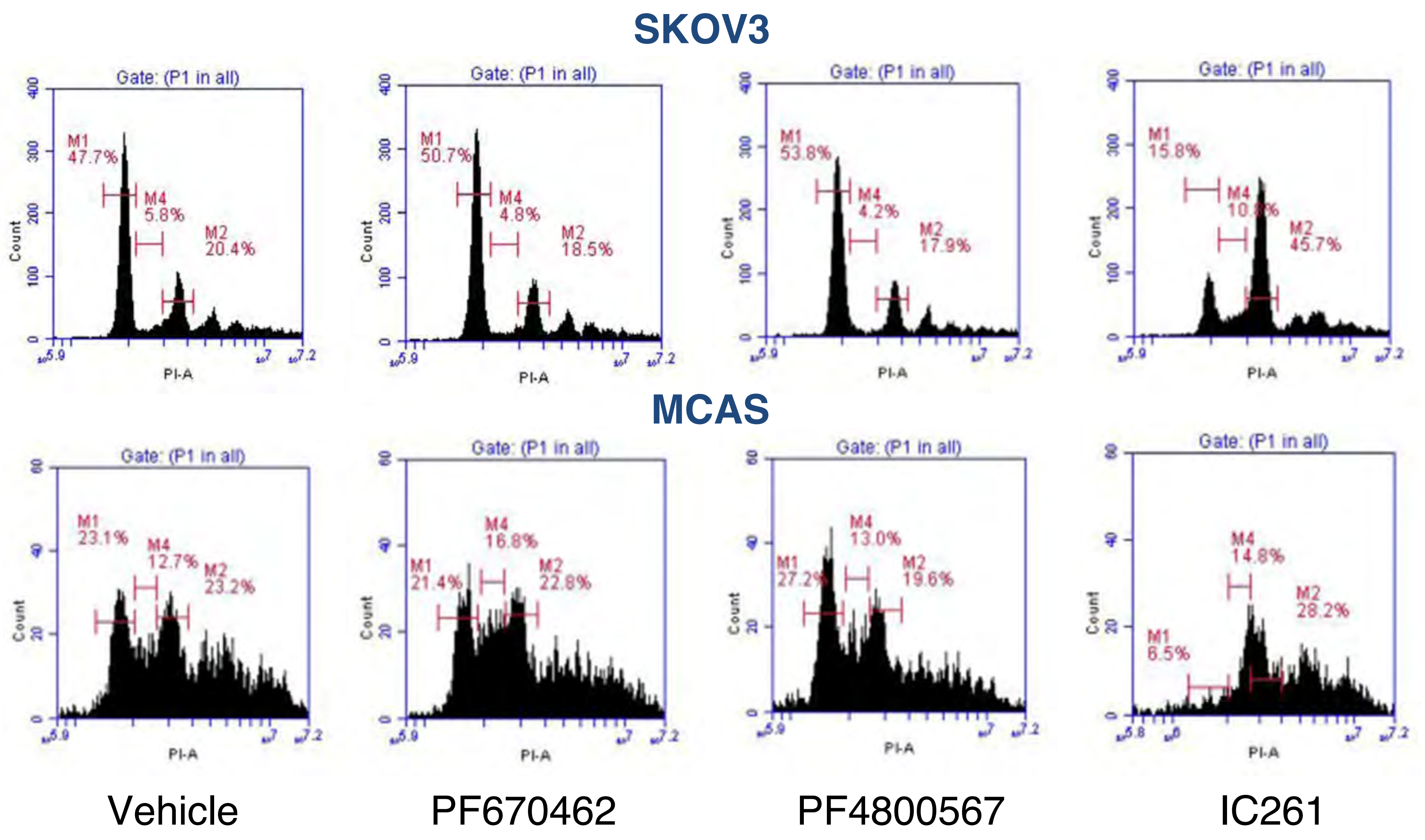

Figure S5. Cell cycle distribution of ovarian cancer cells treated with CKI inhibitors.

Both SKOV3 and MCAS cells were treated with same volumes of DMSO vehicle, 10 μM of PF670462, 50 μM of PF4800567, and 5 μM of IC261 overnight and FACS analyzed.

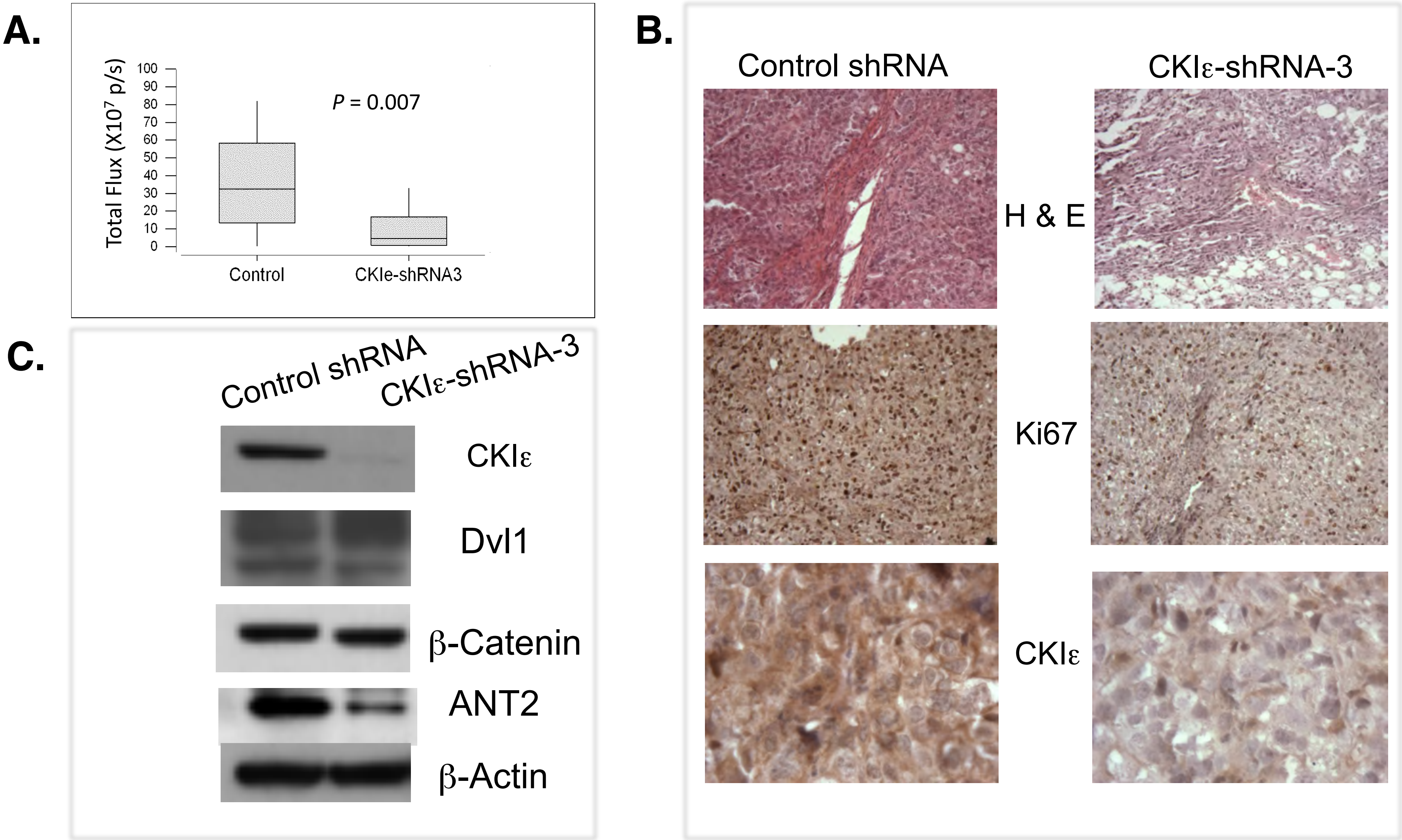

Figure S6. Characterization of xenograft tumors.
A. Boxplot of tumor bioluminescence data. **B.** Immunohistochemical staining of xenograft tumors. **C.** Western blot analysis of the tumor lysates for Wnt signaling proteins and ANT2.

1. Prohibitin

UPTR:Q53FV0_HUMAN Q53fv0 homo sapiens (human). prohibitin variant (fragment). 6/2006 [MASS=29803]

MAAKVFESIG KFGLALAVAG GVVNSALYNV DAGHRAVIFD RFRGVQDIVV GEGTHFLIPW
VQKPIIFDCR SRPRNVPVIT GSK**DLQNVNI TLR**ILFRPVA SQLPR**IFTSI GEDYDERVLP**
SITTEILKSV VAR**FDAGELI TQR**ELVSRQV SDDLTERAAT FGLILDDVSL THLTFGKEFT
EAVEAK**QVAQ QEAER**ARFVV EKAEQQKK**AA IISAEGDSK**A AELIANSLAT AGDGLIKLRK
LEAAEDIAYQ LSRSRNITYL PAGQSVLLQL PQ

2. Adenine nucleotide translocator 2

UPSP:ADT2_HUMAN P05141 homo sapiens (human). adp/atp translocase 2 (adenine nucleotide translocator 2) (ant 2) (adp,atp carrier protein 2) (solute carrier family 25 member 5) (adp,atp carrier protein, fib [MASS=32764]

TDAAVSFAKD FLAGGVAAAI SKTAVAPIER VKLLLQVQHA SKQITADKQY KGIIDCVVRI
PK**EQGVLSFW R**GNLANVIRY FPTQALNFAF KDKYKQIFLG GVDKRTQFWR YFAGNLASGG
AAGATSLCFV YPLDFARTRL AADVGKAGAE REFRGLGDCL VKIYKSDGIK GLYQGFNVSV
QGIIIYR**AAY FGIYDTAK**GM LPDPKNTHIV ISWMIAQTVT AVAGLTSYPF DTVRRRMMMQ
SGRKGTDIMY TGTLDCWRKI ARDEGGKAFF KGAWSNVLRG MGGAFVLVLY DEIKKYT

3. Annexin A2

UPSP:ANXA2_HUMAN P07355 homo sapiens (human). annexin a2 (annexin ii) (lipocortin ii) (calpactin i heavy chain) (chromobindin-8) (p36) (protein i) (placental anticoagulant protein iv) (pap-iv). 7/2006 [MASS=38473]

STVHEILCKL SLEGDHSTPP SAYGSVKAYT NFDAER**DALN IETAIK**TK**GV DEVTIVNILT**
NRSNAQR**QDI AFAYQR**RTKK ELASALKSAL SGHLETVILG LLK**TPAQYDA SELK**ASMKGL
GTDEDSLIEI ICSR**TNQELQ EINR**VYKEMY K**TDLEKDIIS DTSGDFRK**LM VALAKGR**RAE**
DGSVIDYELI DQDARDLYDA GVKRKGTDVP KWISIMTERS VPHLQKVFDR YKSYSPYDML
ESIRKEVKGD LENAFLNLVQ CIQNKPLYFA DRLYDSMKGK GTRDKVLIRI MVSRSEVDML
KIRSEFKRKY GK**SLYYYIQQ DTK**GDYQKAL LYLCGGDD

4. Casein Kinase I epsilon

UPSP:KC1E_HUMAN P49674 homo sapiens (human). casein kinase i isoform epsilon (ec 2.7.11.1) (cki-epsilon) (ckie). 7/2006 [MASS=47315]

MELRVGNKYR LGRKIGSGSF GDIYLGANIA SGEEVAIKLE CVKTKHPQLH IESKFYKMMQ
GGVGIPSIKW CGAEGDYNVM VMELLGPSLE DLFNFCSRKF SLKTVLLLAD QMISRIEYIH
SKNFIHRDVK PDNFLMGLGK KGNLVYIIDF GLAKKYRDAR THQHIPYREN KNLTGTARYA
SINTHLGIEQ SRRDDLESLG YVLMYFNLGS LPWQGLKAAT KRQKYERISE KKMSTPIEVL
CKGYPSEFST YLNFCRSLRF DDKPDYSYLR QLFRNLFHRQ GFSYDYVFDW NMLKFGAARN
PEDVDRERRE HEREERMGQL RGSATRALPP GPPTGATANR LR**SAAEPVAS TPASR**IQPAG
NTSPRAISRV DRERKVSMRL HR**GAPANVSS SDLTGR**QEVS RIPASQTSVP FDHLGK

Figure S7. Peptide mass analysis results of the Prohibitin, ANT2, Annexin A2, and CKIε proteins.

The identified peptide sequences matched to each protein are underlined.

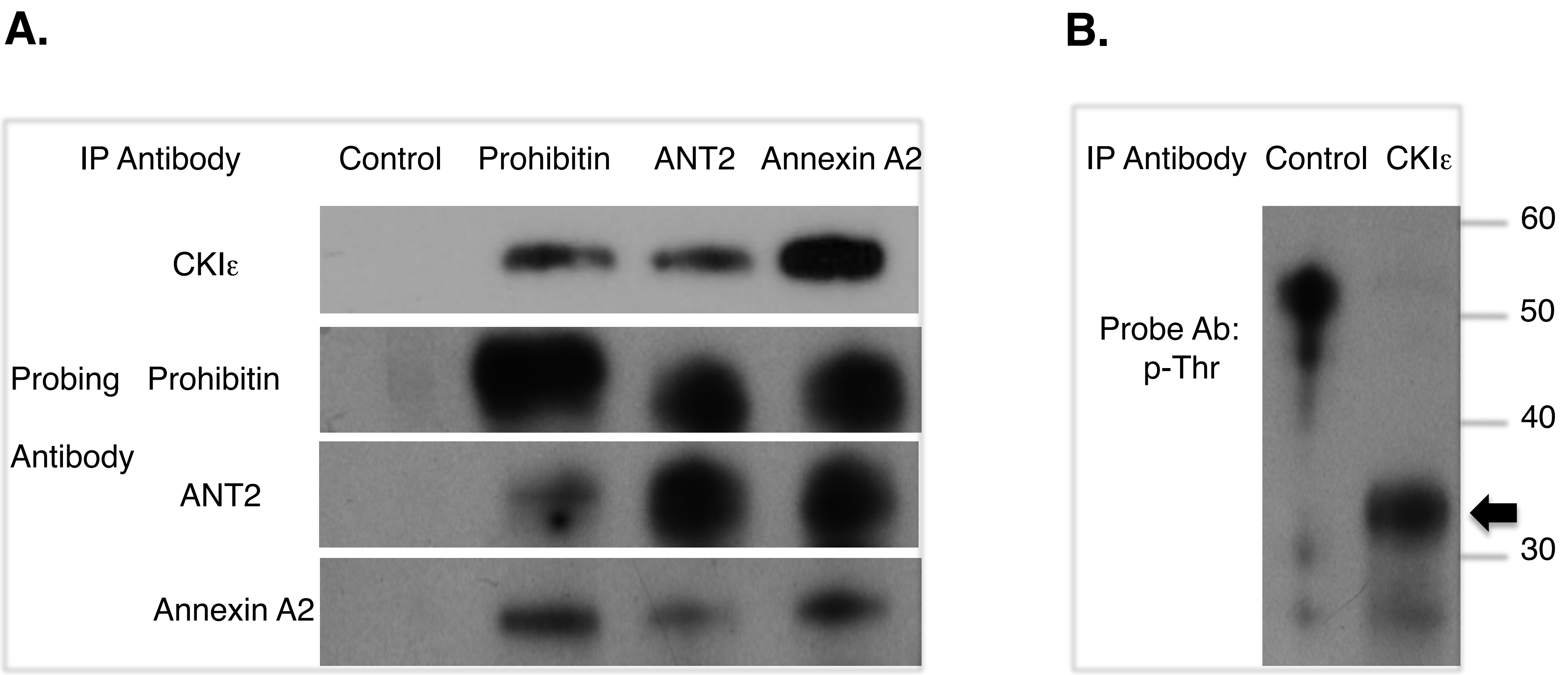

Figure S8. Reciprocal immunoprecipitation assays and phosphorylation status of immunoprecipitated proteins.

A. Immunoprecipitation reactions performed using antibodies specific to Prohibitin, ANT2, and Annexin A2, respectively, were electrophoresed in parallel lanes. After transfer, the membrane was probed with antibodies for CKIε and the three mitochondrial proteins, respectively. **B.** MCAS cell lysates were immunoprecipitated using a control antibody and the CKIε antibody, respectively. The membrane was then probed with an antibody specific to phospho-threonine amino acid. The bands on the control lane were from the heavy and light chains of the antibody recognized by the secondary antibody. The phosphorylated protein band was marked by a block arrow.

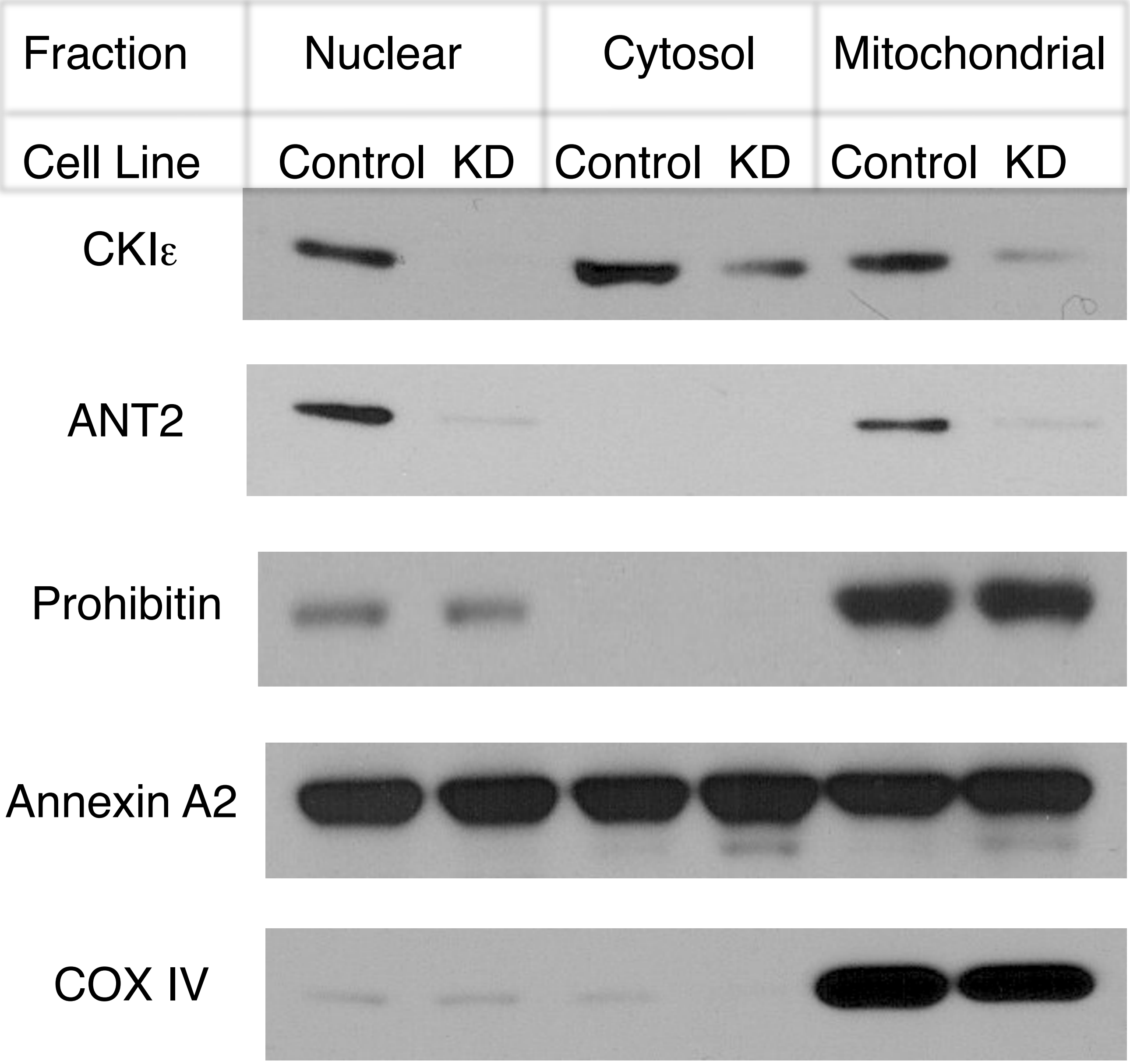

Figure S9. Subcellular distribution of CKIε and the three mitochondrial proteins in the control and CKIε knockdown (KD) cell lines.

Extracts prepared from the nuclear, cytosolic, and mitochondrial fractions of Control shRNA and CKIε shRNA3 knockdown cell lines were electrophoresed and analyzed with antibodies specific to CKIε and the three mitochondrial proteins. COX IV was used as the marker for the mitochondrial fraction.

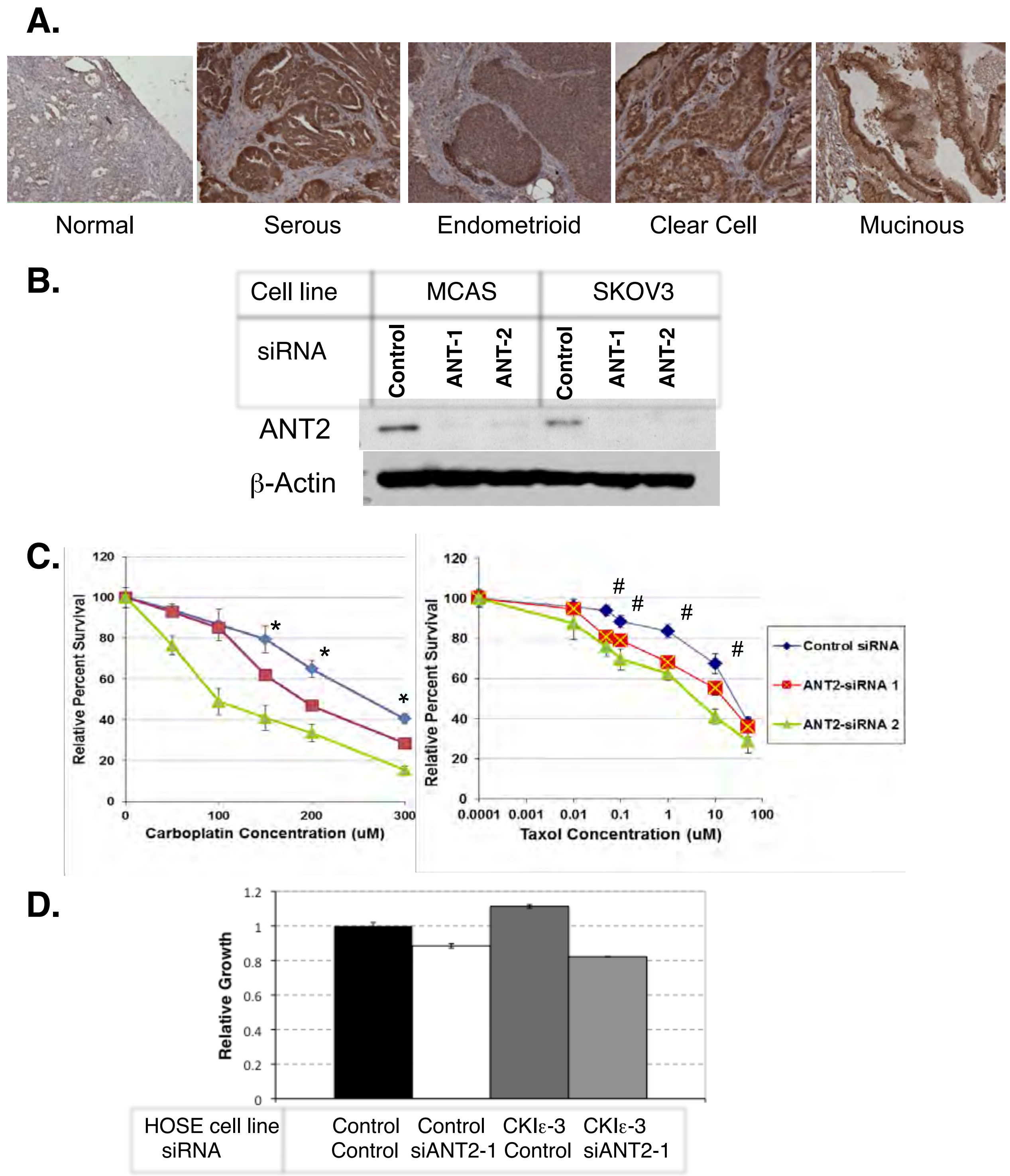

Figure S10. ANT2 mediates the effects of CKIε on ovarian cells.
A. Representative figures of immunohistochemical staining of normal ovaries and ovarian tumor tissues for ANT2. **B.** Western blot analysis to show the knockdown of ANT2 expression in MCAS and SKOV3 cells using ANT2-specific siRNAs. **C.** Cytotoxicity assays to evaluate the sensitivity of control and ANT2 knockdown SKOV3 cells to carboplatin and paclitaxel. *, $P < 0.045$; $^{\#}$, $P < 0.035$. **D.** Relative growth of Control HOSE and CKIε-overexpressing HOSE cell line-3 (CKIε-3) after a mock or an ANT-2 siRNA-1 transfection determined by MTT assay. The growth rates are relative to the control HOSE line with mock transfection.

SI SUPPLEMENTAL TABLES

Table S1. Means and Medians for Survival Time (Positive CKIε versus Negative CKIε)

Tumor type	CKIε status	Mean[a]				Median			
		Estimate	Std. Error	95% Confidence Interval		Estimate	Std. Error	95% Confidence Interval	
				Lower Bound	Upper Bound			Lower Bound	Upper Bound
Non-Serous	Negative	91.620	25.498	41.645	141.596	50.000	19.258	12.254	87.746
	Positive	77.221	21.435	35.209	119.232	35.000	13.370	8.795	61.205
	Overall	87.603	18.636	51.076	124.129	41.370	11.251	19.319	63.421
Serous	Negative	107.471	27.856	52.874	162.069	162.000	.000	.	.
	Positive	45.999	14.772	17.046	74.951	14.730	3.114	8.626	20.834
	Overall	62.442	14.444	34.131	90.753	17.000	5.546	6.130	27.870
Overall	Overall	76.277	12.467	51.843	100.712	28.000	7.549	13.205	42.795

a. Estimation is limited to the largest survival time if it is censored.

Table S2. Log rank test for comparing survival (Positive CKIε versus Negative CKIε)

Tumor type	Chi-Square	Degrees of freedom	P-value
Non-Serous	0.530	1	0.467
Serous	4.376	1	0.036
